# Low-Cost NIR Spectroscopy Versus NMR Spectroscopy for Liquid Manure Characterization

**DOI:** 10.3390/s25216745

**Published:** 2025-11-04

**Authors:** Mehdi Eslamifar, Hamed Tavakoli, Eiko Thiessen, Rainer Kock, Peter Lausen, Eberhard Hartung

**Affiliations:** 1 Institute of Agricultural Engineering, Max-Eyth-Straße 6, Kiel University, 24118 Kiel, Germany; ethiessen@ilv.uni-kiel.de (E.T.); rkock@ilv.uni-kiel.de (R.K.); ehartung@ilv.uni-kiel.de (E.H.); 2 Department of Agromechatronics, Leibniz Institute for Agricultural Engineering and Bioeconomy e.V. (ATB), Max-Eyth-Allee 100, 14469 Potsdam, Germany; htavakoli@atb-potsdam.de; 3 Landwirtschaftskammer Schleswig-Holstein, Grüner Kamp 15-17, 24768 Rendsburg, Germany; plausen@lksh.de

**Keywords:** feature selection methods, liquid manure, machine learning, nuclear magnetic resonance, near-infrared spectroscopy, slurry, two- and three-band indices

## Abstract

Accurate characterization of liquid manure properties, such as dry matter (DM), total nitrogen (TN), ammonium nitrogen (NH_4_-N), and total phosphorus (TP), is essential for effective nutrient management in agriculture. This study investigates the use of near-infrared spectroscopy (NIRS) within the 941–1671 nm range, combined with advanced pre-processing and machine learning techniques to accurately predict the liquid manure properties. The predictive accuracy of NIRS was assessed by comparison with nuclear magnetic resonance (NMR) spectroscopy as a benchmark method. A number of 51 liquid manure samples were analyzed in the laboratory for the reference manure properties and scanned with NIRS and NMR. The NIR data underwent spectral pre-processing, which included two- and three-band index transformations and feature selection. Partial least squares regression (PLSR) and LASSO regression were employed to develop calibration models. According to the results, using cohort-tuned models, NIRS showed fair predictive accuracy for DM (R^2^ = 0.78, RPD = 2.15) compared to factory-calibrated NMR (R^2^ = 0.68, RPD = 0.81). Factory-calibrated NMR outperformed for chemical properties, with R^2^ (RPD) of 0.89 (1.74) for TN, 0.97 (5.70) for NH_4_-N, and 0.95 (2.64) for TP, versus NIRS’s 0.66 (1.68), 0.84 (2.45), and 0.84 (2.51), respectively. In this study with 51 samples, two- and three-band indices significantly enhanced NIRS performance compared to raw data, with R^2^ increases of 34%, 57%, 25%, and 33% for DM, TN, NH_4_-N, and TP, respectively. Feature selection efficiently reduced NIR spectral dimensionality without compromising the prediction accuracy. This study highlights NIRS’s potential as a portable tool for on-site manure characterization, with NMR providing superior laboratory validation, offering complementary approaches for nutrient management.

## 1. Introduction

Manure plays an important role in agricultural production as it enriches soil fertility, improves soil structure, and boosts plant nutrient availability [1,2]. It includes organic material and nutrients that can serve as a soil supplement, decreasing the need for synthetic fertilizers. Manure aids in the retention of soil organic carbon and nitrogen, crucial for mitigating climate change and ensuring food security [3]. In spite of all the advantages mentioned, it is essential to apply manure to agricultural fields in moderation and according to the soil’s needs to avoid excessive application, which can potentially harm plants and pollute the environment [4,5]. Therefore, in addition to soil testing, analyzing manure for its nutrient content, including total nitrogen, ammonium, phosphorus, and potassium, is important for effective nutrient management planning (NMP). NMP ensures optimal nutrient application to fields, maximizing crop yields while minimizing environmental nutrient loss [6].

Traditional nutrient characterization in manure relies on wet chemistry analysis, which is costly, time-consuming, and often subject to inter-laboratory variability. Alternative techniques, such as near-infrared spectroscopy (NIRS) and nuclear magnetic resonance (NMR) spectroscopy, offer rapid, non-destructive analysis for assessing manure composition. However, NIRS and NMR also face reproducibility challenges, including calibration stability and standardization across devices or settings, which can affect result consistency [7]. Despite these limitations, NIRS’s affordability and portability, and NMR’s high precision for chemical properties, make them promising tools for addressing the practical constraints of wet chemistry in on-farm nutrient management [8,9].

Recent studies have expanded NIRS applications to various manure types, including pig slurry. For example, Gogé et al. provided a dataset of 332 samples from cattle, poultry, and pig manure analyzed with NIRS, demonstrating its versatility across livestock types [10]. Additionally, low-field NMR has been evaluated for manure nutrient prediction, with Feng et al. reporting good correlations for total solids, total nitrogen, and ammonium nitrogen in dairy manure using a low-field NMR sensor [11].

NMR is used to analyze the molecular structure and composition of manure by detecting the behavior of atomic nuclei in a magnetic field. This technique is particularly effective for identifying specific compounds in manure, which is crucial for understanding nutrient availability [12]. NMR provides detailed molecular-level information, and the sample remains intact for further analysis, which is beneficial for additional testing [13]. Low-field NMR, in particular, has shown higher precision in measuring ammonium and total nitrogen compared to standard wet chemistry methods, although it is slightly less precise for total phosphorus measurements [11,14]. However, NMR requires expensive equipment and expert knowledge [11] and it may be less effective for elements with low natural abundance or magnetic moments [12]. NMR’s factory calibrations, often based on diverse but limited samples (e.g., 16 in prior validations), provide high precision but may require dataset-specific adjustments for optimal accuracy.

NIR spectroscopy, on the other hand, employs near-infrared light to determine the nutrient composition of manure by measuring the amount of light reflected or absorbed. This technique has been widely used for analyzing manure moisture, nitrogen, and organic matter content due to its rapid and non-destructive principle. It provides quick results, making it ideal for routine use [15] and enabling farmers and researchers to analyze large volumes of manure samples efficiently [16,17]. This rapid analysis is crucial for real-time decision-making, especially during manure application in the field, where timely nutrient information can optimize fertilizer application [15]. However, achieving accurate results relies heavily on the proper calibration of the spectral data to the laboratory-measured reference data, which can be user-dependent [18,19]. NIR spectroscopy employs near-infrared light to assess manure nutrient composition by measuring reflectance or absorbance, offering rapid, non-destructive analysis compared to wet chemistry. However, the calibration methodology in this study, involving 75 spectra per subsample with repositioning (Section 2.2.2), increases measurement time, limiting its practicality for routine on-farm use. Simplified protocols with fewer scans could enhance speed for real-time applications, making NIRS a promising tool for preliminary nutrient screening despite calibration complexities [17].

Despite its lower accuracy compared to techniques like NMR, NIRS is valuable for manure nutrient characterization due to its unique advantages. NIRS equipment is generally more affordable and easier to maintain than other spectroscopic techniques like NMR. This lower cost makes it accessible for routine use on farms and in small research laboratories, allowing for widespread adoption and frequent analysis [20]. This study compares cohort-tuned NIRS models (calibrated on 51 samples) with factory-calibrated NMR, discussing calibration limitations and their impact on generalizability.

The precision of calibration in NIRS for measuring components of liquid manure is influenced by several parameters, including the choice of pre-processing methods. Techniques such as derivatives, multiplicative scatter correction (MSC), Savitzky–Golay smoothing, and standard normal variate (SNV) have been commonly applied to reduce systematic noise, address baseline shifts, and mitigate scattering effects [21]. These pre-processing steps enhance the quality of spectral data, ensuring better alignment between spectral features and the chemical properties of manure during calibration. Recently, index-based transformations, particularly dual- and triple-wavelength indices, were used to further improve calibration accuracy [22]. Dual-wavelength indices, such as the simple ratio index (SRI) and normalized difference index (NDI), have demonstrated the potential to improve the prediction of nitrogen and phosphorus in organic matrices by emphasizing critical absorption bands [8]. Three-band indices (TBIs) have been shown to extend this approach by incorporating information from an additional spectral band, allowing for a more comprehensive representation of complex spectral interactions. This approach has been particularly effective in the cases where overlapping spectral features hinder the interpretation of the two-band indices. To the best of our knowledge, no research has been conducted on using TBIs for manure characterization [23].

In the context of manure analysis with NIRS, pre-processing techniques are essential for addressing challenges such as overlapping absorption bands and scattering effects, which are prevalent in heterogeneous organic materials. Horf et al. (2022) [8] reported that the variability in R^2^ values of calibration models for liquid manure properties assessment, ranging from 0.58 to 0.99, was strongly influenced by pre-processing choices and spectral intervals [8]. These findings underscore the necessity of optimizing pre-processing methods to achieve reliable and reproducible results in NIRS-based nutrient analysis.

Calibration of the models from NIR data poses another issue due to the vast size of the spectral dataset. Various feature selection (FS) techniques, such as recursive feature elimination (RFE), hold the potential to address this [24,25,26,27]. Particularly when coupled with robust algorithms, RFE can effectively mitigate model constraints and computational intricacy, rendering it appropriate for datasets with a high number of features.

NMR measurements were conducted using the TveskaegTM Benchtop NMR analyzer with factory-calibrated models, a controlled chamber temperature of 39 °C, an operating temperature range of 18–28 °C, and analysis times of 1–24 min, depending on the property (Section 2.5). These conditions ensure NMR’s high precision for molecular-level analysis, complementing NIRS’s portability for on-site applications.

This study set out to explore alternative strategies for enhancing the predictive ability of spectroscopic techniques in the determination of properties of liquid manure. To deal with the high dimensionality of near-infrared (NIR) data, three feature selection techniques, e.g., recursive feature elimination with support by support vector machine (RFE-SVM), recursive feature elimination with support by multiple linear regression (RFE-MLR), and the least absolute shrinkage and selection operator (LASSO) were implemented, and their effect on the accuracy of prediction was investigated. In addition, the usefulness of dual-wavelength index transformations, involving the simple ratio index (SRI), the normalized difference index (NDI), as well as three-band index transformations (TBIs), was also investigated with regard to their potential for enhancing the accuracy of prediction. Finally, the capability of NIR spectroscopy was compared with that of nuclear magnetic resonance (NMR) spectroscopy for the prediction of the relevant properties of liquid manure.

This study compares cohort-tuned NIRS models (calibrated on 51 samples) with factory-calibrated NMR, discussing calibration limitations and their impact on generalizability. The comparison is framed as practical rather than methodologically equivalent, evaluating NIRS’s affordability and portability for on-farm screening against NMR’s precision for laboratory validation, offering complementary tools for nutrient management. This approach highlights their respective strengths in real-world agricultural applications, where rapid, cost-effective analysis (NIRS) and high-precision laboratory measurements (NMR) serve distinct but complementary roles in optimizing nutrient management planning.

The novelty of this study lies in the pioneering use of three-band indices (TBIs) for liquid manure characterization, significantly enhancing NIR prediction accuracy, and a direct comparison of low-cost, portable NIR spectroscopy (941–1671 nm) with NMR, evaluating their trade-offs for on-farm nutrient management. Unlike prior studies using wider NIR ranges or mid-IR for higher accuracies (e.g., Zimmermann et al., and Horf et al.) [8,28], this work focuses on practical, resource-constrained applications, advancing affordable manure analysis (Cabassi et al.) [29].

## 2. Material and Methods

### 2.1. Manure Sampling

A total of 51 organic fertilizer samples, including 31 cattle slurry samples, 8 pig slurry samples, and 12 biogas residue samples and combinations, were collected from 13 farms in Northern Germany between February and March 2021. For sampling, a mobile manure test bench was built and mounted on a trailer. The sampling process involved:

- Manure mixing: Liquid manure in the storage facility was thoroughly mixed using a mixer (Thermomix TM31, Cloyes-les-Trois-Rivières, France) to ensure uniformity;
- Sample extraction: A closed system with input and output lines transferred liquid manure from the lagoon to a 6 m^3^ tank using a vacuum pump;
- Homogenization: The manure circulated continuously for 10 min within the tank using a rotary lobe pump (Vogelsang R136-420S, Germany) for complete blending and homogenization;
- Representative sample collection: A dedicated valve extracted a sample from the closed system into a 100 L barrel containing approximately 60 L of pre-mixed manure;
- Enhanced homogeneity: The sample underwent further mixing within the barrel using a macerator (Dynamic SMX 800 T Supermixer, 11,000 rpm, 1000 W, Kehl Auenheim, Germany) to ensure a representative sampling;
- Subsampling and preservation: After the homogenization, three 1 L aliquots were collected for laboratory analysis, and for scanning with NIR and NMR sensors. These subsamples were promptly cooled and preserved during transport. At the end of the day, all samples were frozen at −21 °C to store for further analysis.

### 2.2. Data Acquisition

#### 2.2.1. Reference Analysis

The all liquid manure samples were analyzed (wet-chemistry reference) parallel in three different the laboratories (Supplementary Figure S1) for total nitrogen (TN, g/kg), total phosphorus (TP, g/kg), ammonium nitrogen (NH_4_-N, g/kg), and dry matter (DM, %). The selection of TN, TP, NH_4_-N, and DM was based on their critical roles in assessing the nutrient composition and fertilizing potential of liquid manure. TN and NH_4_-N serve as primary indicators of nitrogen availability for plant uptake, with NH_4_-N representing the readily available fraction and TN encompassing the total nitrogen pool. TP is essential for evaluating phosphorus content, which is vital for fertilization planning and mitigating environmental risks such as eutrophication. DM was included due to its influence on nutrient concentration and the physical handling properties of manure. These parameters are widely regarded as fundamental in manure analysis for effective nutrient management (e.g., Horf et al., 2022) [8].

The standard error of the laboratory measurements was calculated from the three replicate analyses per sample conducted at accredited laboratories. The average standard errors were 0.3% for DM, 0.1% for TN, 0.02% for NH_4_-N, and 0.01% for TP [30,31]. These values provide a benchmark for evaluating the performance of the spectroscopic models.

Table 1 presents the methods used for the analyses.

#### 2.2.2. NIR Spectra Acquisition

**Figure 1:**
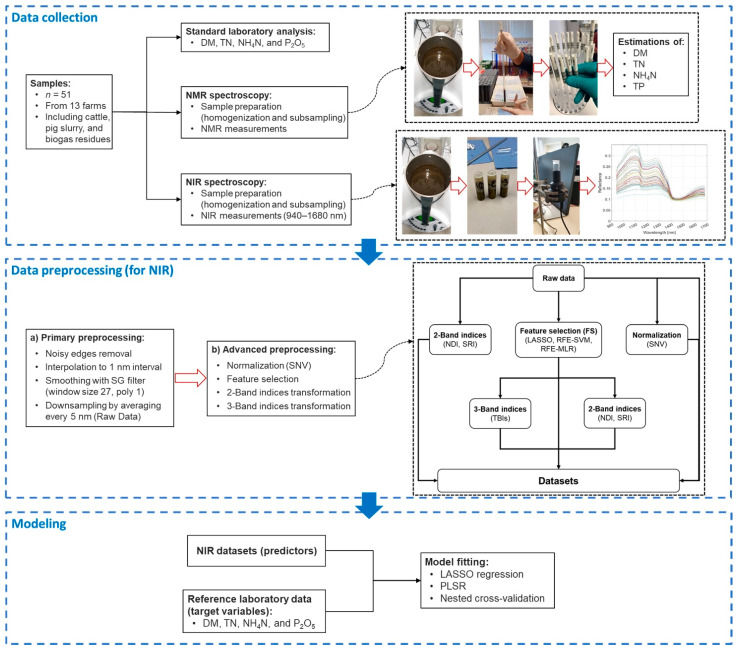
Illustration of the NIR spectroscopy steps.

This study utilized a MicroNIR 1700ES spectrometer (VIAVI Micronir, San Jose, CA, USA) for collecting spectral data from liquid manure samples in a controlled laboratory environment. The spectrometer will hereafter be referred to as VIAVI in this paper. VIAVI operates in the near-infrared (NIR) range of 908–1676 nm, offering a mean resolution of 6.2 nm. It employs a vacuum tungsten light source for illumination. A Spectralon^®^ (VIAVI Micronir, San Jose, CA, USA) plate was used for white referencing, establishing a baseline for reflectance. Dark referencing was performed internally within the instrument. The integration time was set at 10 ms, with 100 scans averaged for each measurement. Figure 1 displays the averaged NIR spectral data, differentiated by substrate.

For the NIR measurements, three separate cuvettes were filled with approximately 7 mL of manure from the same subsample. Before measurements, each cuvette was gently shaken to create five distinct sample surfaces. Five spectra were then collected from each of these surfaces, resulting in a total of 25 spectra per cuvette (five spectra by five surfaces) and 75 spectra per subsample (25 spectra/cuvette by three cuvettes). To account for the variability within the subsample, the average of its 75 individual spectral measurements was calculated, which was then used for subsequent modeling.

#### 2.2.3. Model Validation Enhancements

To provide reliable real-world estimates, NIR models were evaluated with a 20% hold-out test set (*n* = 10 samples, stratified by manure type to preserve diversity). The test set was randomly selected once, with the remaining 80% (*n* = 41) used for training. Metrics (R^2^, RMSE, RPD) were computed on this held-out data. NMR’s pre-calibrated design limited similar splitting, relying on cited external validations.

### 2.3. NIR Spectral Data Processing: Pre-Processing and Feature Selection

#### 2.3.1. Spectral Pre-Processing

Figure 1 presents a comprehensive workflow, encompassing all the steps involved in this study, from sample collection to model development. It highlights the exploration of 50 unique combinations of pre-processing techniques employed to predict the components of liquid manure. The details of these pre-processing combinations are provided in Supplementary Table S1.

Given that VIAVI’s output is presented as pseudo-absorbance (*A*), the data was transformed to reflectance (*R*) according to the following equation:

(1) $$R\;=\;1/10^{A}$$

The NIR reflectance data underwent several pre-processing steps (Figure 1). First, interpolation to a 1 nm interval ensured having regular resolution. Second, spectral edges were adjusted to address low signal-to-noise ratios, resulting in a modified usable range of 941 to 1671 nm. Next, the spectra were smoothed using Savitzky–Golay technique [36] through a first-order polynomial fit with a 13-point window. To lower the dimensionality, data points were averaged every 5 nm, leading to a final set of 146 wavebands (variables). Averaging data points every 5 nm (reducing from ~731 variables at 1 nm resolution to 146 wavebands) was chosen to lower spectral dimensionality, reduce multicollinearity in highly correlated adjacent wavelengths, and improve computational efficiency for modeling (PLSR, LASSO), while aligning with the spectrometer’s mean resolution of 6.2 nm to minimize information loss. Potential loss was evaluated by comparing model performance (R^2^, RMSE, RPD) on raw (1 nm) versus averaged spectra; preliminary tests showed negligible differences (<2% R^2^ drop across properties), confirming retention of key absorption features (e.g., water at 970 nm, N-H at 1500–1550 nm) [37]. Throughout this paper, this dataset will be referred to as the “raw data”. Additionally, standard normal variate (SNV) correction was applied to normalize the spectra by subtracting the mean and dividing by the standard deviation of each spectrum.

This study explored two types of dual-wavelength indices: Normalized difference indices (*NDI*) [38] and simple ratio indices (*SRI*) [22]. These indices were calculated using various combinations of wavebands (*R_i_* and *R_j_*) on both the raw data and subsets identified through feature selection methods. The formulas for the *NDI* and *SRI* calculation are provided below:

(2) $${NDI}_{ij}\;=\;\frac{R_{j}\;-\;R_{i}}{R_{j}\;+\;R_{i}}$$

(3) $${SRI}_{ij}\;=\frac{R_{j}}{R_{i}}$$

where *R* represents the mean reflectance values and *i* and *j* follow the condition: *i* < *j* < = 2…146. Employing this approach for both *NDI* and *SRI* on the raw data resulted in a total of 10,878 unique two-band combinations (variables).

To further explore the potential of *NIR* spectroscopy for liquid manure characterization, this study investigated the use of three-band indices (*TBI*s). All possible combinations of three wavebands were calculated using the following formulas:

(4) $$TBI1=\frac{\left|R_{i}-R_{j}\right|}{R_{k}}$$

(5) $$TBI2=\frac{R_{i}+R_{j}}{R_{k}}$$

(6) $$TBI3=\frac{R_{i}\times R_{j}}{R_{k}}$$

(7) $$TBI4=\frac{\left|R_{i}-R_{j}\right|}{\left(R_{i}+R_{k}\right)}$$

where TBI stands for three-band index, with *R_i_*, *R_j_*, and *R_k_* representing the reflectance values for any three bands within the selected subsets obtained from feature selection, where *i < j*, and *j < k ≤* 3…146.

The pre-processing methods were selected to enhance spectral signals related to manure components (DM, TN, NH_4_-N, TP) by addressing specific challenges in NIR data (Burns & Ciurczak, 2020) [39]. Standard normal variate (SNV) correction normalizes spectra to reduce scattering effects from heterogeneous manure matrices, improving signal clarity for DM and organic matter. Savitzky–Golay smoothing (first-order polynomial, 13-point window) minimizes noise while preserving absorption features, such as water bands (~970 nm, 1450 nm) critical for DM and C-H/N-H bands (~1200 nm, 1500–1550 nm) for TN and NH_4_-N. Two-band indices (NDI, SRI) emphasize key absorption band ratios (e.g., N-H at 1500–1550 nm vs. reference bands) to enhance TN and NH_4_-N signals by mitigating baseline shifts [40,41]. Three-band indices (TBIs) incorporate additional bands to capture complex interactions (e.g., water, C-H, and N-H), improving TP and NH_4_-N predictions by resolving overlapping absorptions in heterogeneous matrices. These mechanistically motivated methods were complemented by exploratory testing to optimize performance across manure types.

#### 2.3.2. Feature Selection

Least Absolute Shrinkage and Selection Operator (LASSO) is a regression method that performs variable selection and regularization to enhance prediction accuracy and model interpretability. It includes a penalty term proportional to the sum of absolute coefficients, encouraging sparsity by setting some coefficients to zero, thus selecting only the most relevant features [42].

This study employed two feature selection techniques: Recursive feature elimination (RFE) and least absolute shrinkage and selection operator (LASSO). RFE was combined with two different machine learning models as its core algorithm—multi-linear regression (MLR) and support vector machine (SVM). These combinations will be referred to as RFE-SVM and RFE-MLR throughout the text.

To identify important features for RFE-MLR, absolute cross-validation accuracy was used to rank features from strongest to weakest. Conversely, RFE-SVM identified the optimal number of features based on the minimization of negative mean squared error. For LASSO, features were ranked based on the absolute values of their corresponding coefficients. The two-band and three-band indices, along with models built using PLSR and LASSO regression, were subsequently calculated directly on the selected feature subsets. The full name and details of these feature selection methods are shown in Supplementary Table S1.

### 2.4. Modeling

#### 2.4.1. Machine Learning Methods

In this study, nested 10-fold cross-validation was employed to evaluate all models, with the dataset of 51 samples divided into 10 folds (~5 samples per fold). In each iteration, approximately 46 samples were used for training and 5 for validation, with the process repeated 10 times to ensure all samples contributed to both calibration and validation. This approach, utilizing inner and outer loops, balanced bias-variance trade-offs, maximized data utilization, and minimized overfitting risks, providing robust performance metrics (R^2^, RMSE, RPD) averaged across validation folds [43]. To mitigate the imbalance in manure types (31 cattle slurry, 8 pig slurry, 12 biogas residues), stratified nested cross-validation was implemented, ensuring proportional representation (~3 cattle, 1 pig, 1 biogas residue per fold). Preliminary results showed consistent performance (R^2^ variation < 6% across types), though the small pig slurry subset (*n* = 8) limits generalizability.

For model optimization, a machine learning pipeline was constructed for partial least squares regression (PLSR) to streamline and standardize the modeling process. This pipeline integrated data standardization (scaling features to zero mean and unit variance) and the PLSR regressor, addressing PLSR’s sensitivity to variable scale in spectral data. Grid search within the inner loop of nested cross-validation optimized hyperparameters: the number of latent variables for PLSR and the regularization parameter (alpha) for LASSO, balancing model complexity and predictive accuracy. For PLSR models (DM, TN, NH_4_-N), the number of latent variables (15 for DM, 20 for TN, 31 for NH_4_-N) was optimized via grid search to maximize predictive performance while capturing complex spectral interactions in heterogeneous manure matrices. For TP, LASSO’s regularization parameter (alpha = 0.1) was similarly optimized, controlling model complexity without latent variables.

Alternative cross-validation strategies, such as leave-one-out cross-validation (LOOCV) and repeated 10-fold cross-validation, were considered but discarded. LOOCV’s high computational cost and overfitting risk in high-dimensional NIR data, along with minimal improvements (<5% R^2^ variation) from repeated cross-validation in preliminary tests, justified the use of nested 10-fold cross-validation for this study [31,44]. To assess model transferability, a supplementary leave-one-group-out (LOGO) cross-validation was performed, holding out entire manure types (e.g., cattle slurry) for testing while training on remaining groups [11]. LOGO results indicated moderate performance drops for pig slurry (R^2^ reduced by 10–15% for TN and NH_4_-N), underscoring the need for larger, more balanced datasets (>100 samples) in future studies to enhance stability and generalizability across manure types.

#### 2.4.2. Model Performance Assessment

Performance of cross-validated models was evaluated using the coefficient of determination (R^2^), root mean squared error (RMSE), and the ratio of performance to deviation (RPD) [45], calculated using the equations below:

(8) $$R^{2}\;=\;1\;-\;\frac{\sum_{i=1}^{n}{\left(y_{i}\;-\;{\hat{y}}_{i}\right)}^{2}}{\sum_{i=1}^{n}{\left(y_{i}\;-\;\overset{-}{y}\right)}^{2}}$$

(9) $$RMSE=\;\sqrt{\;\frac{1}{n}\sum_{i=1}^{n}{\left(y_{i}-{\hat{y}}_{i}\right)}^{2}}$$

(10) $$\overset{-}{y}=\frac{1}{n}\sum_{i=1}^{n}y_{i}$$

(11) $$RPD\;=\frac{SD}{RMSE}$$

(12) $$SD=\sqrt{\frac{\sum_{i=1}^{n}{\left(y_{i}-\bar{y}\right)}^{2}}{n-1}}$$

where *n* is the number of samples; *y_i_* represents the observed value for the target variable *y* of the manure sample *i*; ${\hat{y}}_{i}$ is the predicted value of the sample *i*, $\bar{y}$ denotes the mean of observed values; and *SD* stands for the standard deviation of observed values.

RPD values can be used to interpret the calibration quality according to the following categories [29]:

- Excellent: RPD > 4.0
- Successful: 3.0 ≤ RPD ≤ 4.0
- Useful: 2.2 ≤ RPD ≤ 3.0
- Moderately useful: 1.7 ≤ RPD ≤ 2.2
- Acceptable: 1.5 ≤ RPD ≤ 1.7
- Poor: RPD < 1.5

To determine whether small differences in R^2^ values across pre-processing methods and models are meaningful, paired *t*-tests were conducted to compare performance metrics from nested 10-fold cross-validation, with bootstrap confidence intervals calculated to assess metric stability (Dietterich, 1998) [46]. Significant improvements (*p* < 0.05) are noted in Supplementary Table S2, distinguishing robust enhancements from potential noise in the small dataset (*n* = 51).

#### 2.4.3. Implementation Platform

Python’s scikit-learn library (v3.11) [Python, 2021] was used to build both PLSR and LASSO models. Spectral interpolation, two-band and three-band index calculations were performed using MATLAB software (version 24.1, R2024a; The MathWorks Company, Natick, MA, USA). Savitzky–Golay filtering was applied using Unscrambler software (version 10.5.1). All data analysis and model training were conducted on a single machine with the following specifications: Intel^®^ Core™ i7-9700 CPU @ 3.00 GHz (8 cores), 64 GB RAM, and Windows 10 Education (version 22H2).

### 2.5. NMR Measurements

Low-field nuclear magnetic resonance (NMR) is a non-destructive technique that measures relaxation times of atomic nuclei, particularly hydrogen (1H), in a magnetic field. For manure analysis, low-field NMR correlates these relaxation parameters with nutrient concentrations. The Tveskaeg^TM^ Benchtop NMR analyzer uses pre-calibrated models to predict DM, TN, NH_4_-N, and TP based on NMR signals from manure samples, leveraging the distinct relaxation properties of molecular components [11].

The nuclear magnetic resonance (NMR) measurements were conducted using a Tveskaeg^TM^ Benchtop NMR analyzer (NanoNord A/S, Aalborg, Denmark) equipped with a powerful, shielded magnet that generates a uniform magnetic field. The manufacturer pre-calibrated all parameters (DM, TN, NH_4_-N, and TP) using reference samples with known concentrations, and no additional pre-processing was applied to the NMR data in this study, relying instead on the factory settings for consistency. The system also includes a radio-frequency antenna, digital signal processing units, and a computer for NMR analysis. The manufacturer pre-calibrated all parameters (DM, TN, NH_4_-N, and TP) using reference samples with known concentrations. Before use, the system must be warmed up to a standard operating temperature range of 18–28 °C. The interior measuring chamber maintained a constant temperature of 39 °C. The NMR signal utilizes a range of transmitting frequencies between 2 and 70 MHz.

Before analysis, the 1 L subsamples were homogenized for 30 s using a Vorwerk Thermomix TM31 mixer. The homogenized manure was then transferred into specialized plastic tubes (diameter: 9.2 mm) to a fill level of 42 mm. Three tubes per subsample were prepared, and each tube was measured three times. A sample changer, with 24 slots, automatically loaded the tubes into the NMR measurement chamber. Analysis times were 1 min for DM and TN, and 24 min for NH_4_-N and TP. When tested with a reference sample, the factory-calibrated NMR analyzer demonstrated a precision of ±3% and an accuracy range of 0.1 mg/L to 10 g/L.

The factory calibration, as detailed in validation studies (Sørensen et al., Feng et al.) [11,47], is based on ~16 diverse manure samples (cattle, pig, mink, biogas residues) covering ranges like NH_4_-N 300–4600 mg/L and TN 400–6000 mg/L. Validation involved external lab comparisons, yielding R^2^ > 0.85 for most parameters in dairy manure.

## 3. Results and Discussion

### 3.1. Reference Laboratory Results

The findings from the wet-chemistry reference analyses establish a reliable baseline for understanding the variability in the liquid manure properties. To ensure accuracy and minimize measurement bias, samples were analyzed at three accredited laboratories. Only minimal variation between measurements of the laboratories was observed (Supplementary Figure S1). Consequently, the mean values of the three laboratories were used to build calibration models. The normality of all manure properties was assessed using the Shapiro–Wilk test, and the results confirmed that all variables followed a normal distribution. This confirmation is critical because the statistical techniques used in this study; specifically, PLSR and LASSO, as detailed in Section 2.4.1, benefit from normally distributed response variables (TN, TP, NH_4_-N, DM). Although these methods do not strictly require normality in the predictor variables (spectral data), a normal distribution of the response variables supports assumptions about error distribution and enhances the reliability of performance metrics (e.g., R^2^, RMSE) reported in Section 3.2 and Section 3.3 for NIRS and NMR model evaluation.

Table 2 summarizes the descriptive statistics of the manure properties across the study farms, revealing substantial variability. DM content ranged from 0.86% to 9.68%, with a median of 6.62%. TN and NH_4_-N also exhibited significant differences, with TN ranging from 0.63 to 6.74 g/kg (median: 3.43 g/kg) and NH_4_-N ranging from 0.38 to 5.09 g/kg (median: 1.98 g/kg). TP varied between 0.26 and 4.10 g/kg, with a median of 1.38 g/kg. These ranges reflect the diverse manure types (cattle slurry, pig slurry, and biogas residues) sampled from 13 farms in Northern Germany. While this variability aligns with manure composition reported in German studies (e.g., Horf et al., 2022) [8], it primarily represents conditions in Northern Germany, where these samples were collected. Regional differences in livestock management, climate, and soil types across Germany (e.g., drier conditions in southern regions) may lead to variations not fully captured here. Nevertheless, the inclusion of common manure types suggests these results are reasonably representative of Northern German agricultural practices, though additional sampling from other regions (e.g., southern Germany with drier conditions) would strengthen broader applicability across diverse German contexts.

The reference laboratory results (Table 2) reveal considerable variability in the liquid manure properties across the 51 samples, reflecting the diverse composition of cattle slurry, pig slurry, and biogas residues from Northern German farms. The wide ranges, e.g., DM from 0.86% to 9.68% and TN from 0.63 to 6.74 g/kg, are consistent with the heterogeneity reported in prior studies (e.g., Horf et al., 2022) [8], driven by factors such as animal diet, storage conditions, and processing methods. The normal distribution of these properties, confirmed by the Shapiro–Wilk test, supports the applicability of parametric statistical methods like PLSR and LASSO in subsequent modeling, enhancing confidence in the calibration process. Minimal inter-laboratory variation (Supplementary Figure S1) underscores the reliability of the wet-chemistry analyses as a reference standard, providing a robust baseline for comparing NIRS and NMR predictions. However, the high coefficients of variation (e.g., 0.52 for TP) suggest challenges for spectroscopic techniques, as such variability may complicate the detection of subtle spectral signals, particularly for chemical properties like TP.

In this study, a total of 51 liquid manure samples were analyzed, comprising 31 cattle slurry samples, 8 pig slurry samples, and 12 biogas residue samples. The relatively small number of pig slurry samples (*n* = 8) may limit the model’s ability to capture the full variability specific to pig slurry. Previous research has employed larger sample sizes for pig slurry, such as 12 samples in a study by Finzi et al. [48], and comprehensive datasets with 490 samples from various livestock types by Morvan et al. [49]. Despite this limitation, the models developed in the current study demonstrated satisfactory performance for the properties analyzed. To enhance the stability and generalizability of the models, future research should aim to include a larger and more balanced dataset, encompassing a greater number of pig slurry samples.

To enhance transparency, inter-laboratory standard deviations for wet chemistry measurements across three accredited laboratories were calculated and are reported in Supplementary Table S1. These values (e.g., 0.28% for DM, 0.09 g/kg for TN, 0.018 g/kg for NH_4_-N, 0.009 g/kg for TP) confirm minimal variation, supporting the reliability of the reference dataset used for calibration models [50]. Low inter-laboratory variability strengthens confidence in the accuracy of the reference values for DM, TN, NH_4_-N, and TP.

### 3.2. NIRS Results

**Figure 2:**
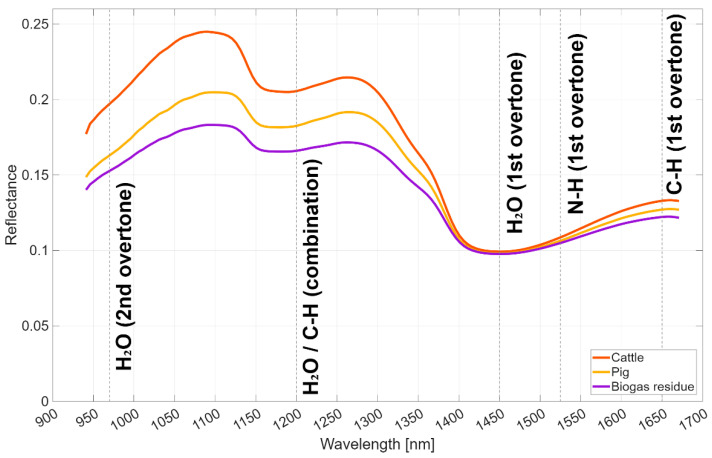
Average NIR spectra of cattle slurry, pig slurry, and biogas residue samples, with key absorption bands annotated. The spectra cover the range of 941–1671 nm, with annotations for water absorption bands at approximately 970 nm (third overtone), 1200 nm (combination), and 1450 nm (first overtone); C-H bonds at 1200 nm (second overtone) and 1650–1671 nm (first overtone); and N-H bonds at 1500–1550 nm (first overtone), which are relevant for predicting DM, TN, and NH_4_-N [51].

**Figure 3:**
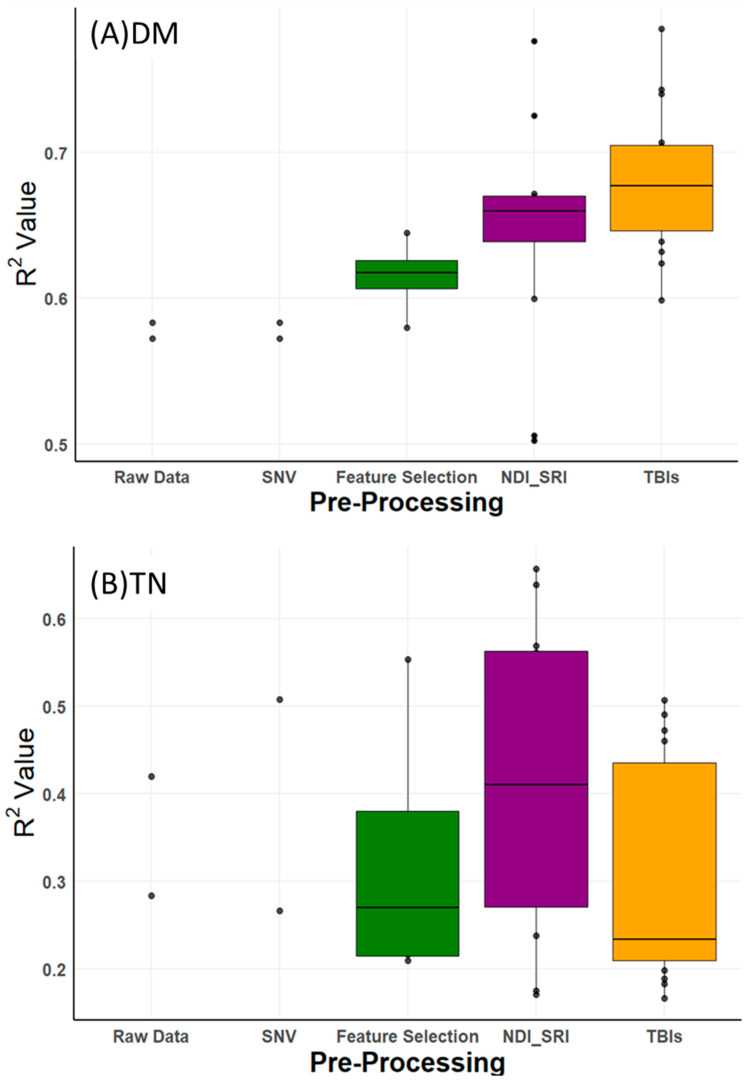

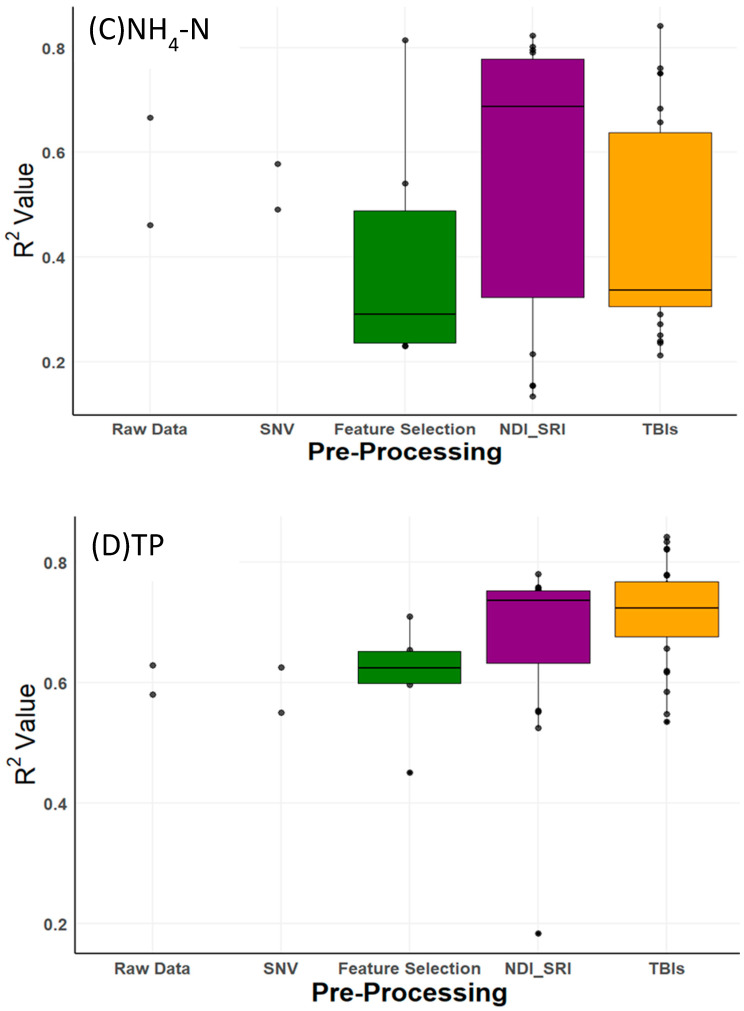
Boxplots presenting performance of NIRS for predicting (**A**) dry matter (DM), (**B**) total nitrogen (TN), (**C**) ammonium nitrogen (NH_4_-N), and (**D**) total phosphorus (TP), by applying different pre-processing and two calibration methods.

This section presents the results obtained using NIRS for predicting liquid manure properties, including dry matter (DM), total nitrogen (TN), ammonium nitrogen (NH_4_-N), and total phosphorus (TP). A total of 48 different pre-processing combinations were applied to the NIR spectra, and their impact on prediction accuracy was evaluated, with results detailed in Figure 3, Table 3, and Supplementary Table S2. Figure 2 displays the averaged NIR spectral data, differentiated by substrate.

The top-performing models for each property, based on Table 3, were: TBI3 with RFE-MLR and PLSR for DM (R^2^ = 0.78, RPD = 2.2), SRI on raw data with PLSR for TN (R^2^ = 0.66, RPD = 1.7), TBI3 with RFE-SVM and PLSR for NH_4_-N (R^2^ = 0.84, RPD = 2.5), and TBI1 with LASSO for TP (R^2^ = 0.84, RPD = 2.5).

As shown in Figure 3, boxplots of R^2^ values illustrate the variability in prediction accuracy across these pre-processing methods. Compared to raw data, pre-processing improved R^2^ values for DM by 0.20 (from 0.58 to 0.78), TN by 0.24 (from 0.42 to 0.66), NH_4_-N by 0.17 (from 0.67 to 0.84), and TP by 0.21 (from 0.63 to 0.84). However, these improvements were not uniform. For DM, NH_4_-N, and TP, three-band indices (TBIs) consistently drove the highest accuracies (Table 3), reflecting their ability to enhance spectral feature extraction. For TN, the best result (R^2^ = 0.66) came from the two-band SRI on raw data, not TBI, indicating that pre-processing benefits vary by property and that TN prediction remains less accurate overall. The ‘raw data’ and ‘SNV’ categories in Figure 3 each show only two R^2^ values, representing the PLSR and LASSO models applied directly to these datasets, unlike index-based methods (e.g., TBI, NDI) that generate multiple combinations. The slight differences between these values (e.g., raw data, RPD of 1.2–1.4, SNV, RPD of 1.3–1.5 for most properties, per Supplementary Table S2) arise from PLSR’s strength in capturing latent structures versus LASSO’s sparsity-driven feature selection.

Table 3 summarizes the performance of top-performing models with RPD > 1.7 (‘moderately useful’ or better, per Section 2.4.2), as this threshold retains a broader set of viable models compared to RPD ≥ 2.2 (‘useful’), which would exclude most TN results. Across the 48 different combinations tested, many yielded RPD values above and below 2.0 (Supplementary Table S2), with raw data and SNV often falling below 1.5 (‘poor’), underscoring the value of pre-processing. For DM, the highest accuracy (R^2^ = 0.78, RMSE = 1.02%, RPD = 2.2) was obtained using TBI3 with RFE-MLR and PLSR. For NH_4_-N, the optimal result (R^2^ = 0.84, RMSE = 0.42 g/kg, RPD = 2.5) came from TBI3 with RFE-SVM and PLSR. For TP, TBI1 with LASSO achieved the best performance (R^2^ = 0.84, RMSE = 0.35 g/kg, RPD = 2.5). For TN, SRI on raw data with PLSR yielded the highest accuracy (R^2^ = 0.66, RMSE = 0.70 g/kg, RPD = 1.7), though this remains lower than other properties and only ‘acceptable’ (RPD < 1.7). This modest performance, compared to NMR (R^2^ = 0.89) and prior studies (e.g., Zimmermann et al., R^2^ = 0.96), underscores NIRS’s current limitation for precise TN prediction, likely due to the narrow spectral range (941–1671 nm) and smaller sample size (51 vs. 206).

These parameters were chosen to maximize predictive performance while preventing overfitting, as validated by the cross-validation results.

To quantify variability in the top-performing NIRS models and address calculation discrepancies, Table 4 presents the mean, standard deviation (SD), and overall out-of-fold (OOF) metrics for R^2^, RMSE, and RPD across nested 10-fold cross-validation for predicting dry matter (DM), ammonium nitrogen (NH_4_-N), total phosphorus (TP), and total nitrogen (TN), using the optimized models from Table 3 (e.g., FS_MLR_TBI3 for DM, SRI_ALL for TN, FS_SVM_TBI3 for NH_4_-N, FS_LASSO_TBI1 for TP).

Table 4 confirms the stability of the optimized models, with low variability (e.g., SD R^2^ = 0.12–0.18, SD RPD = 0.25–0.35) due to preprocessing techniques like TBIs and feature selection (Section 3.2.3). The 95% CI for R^2^ (e.g., DM: [0.69, 0.87], TN: [0.55, 0.77], calculated as Mean ± 1.96 × SD/√10) reflects consistent performance across folds. The minor differences between Mean R^2^ and Overall R^2^ (OOF) (e.g., 0.01 for TN) are expected due to small validation sets (~5 samples per fold), with OOF metrics providing a robust estimate of generalizability. Compared to raw data models (RPD < 1.5, Section 3.2.3), Table 4’s metrics align with Table 3, confirming the effectiveness of preprocessing (e.g., 34% R^2^ increase for DM). These findings highlight the need for larger datasets (>100 samples) and broader spectral ranges (beyond 941–1671 nm) to further stabilize TN predictions, as discussed in Section 3.2.3.

#### 3.2.1. Performance of Different Calibration Models

**Figure 4:**
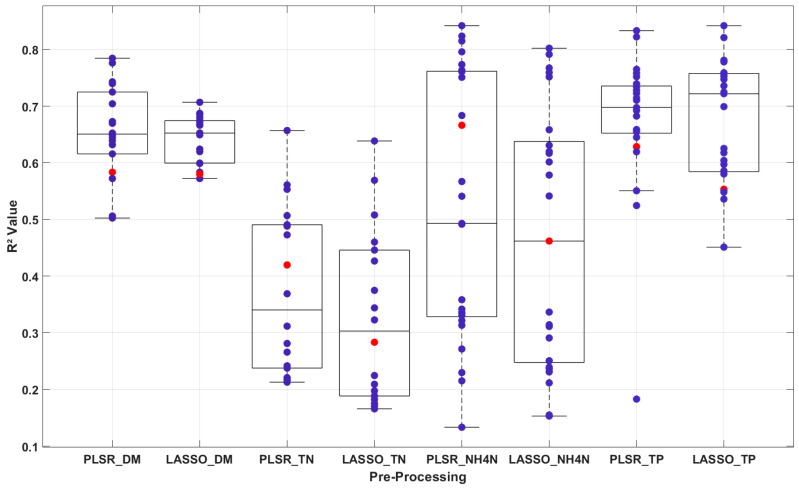
Comparison between partial least square regression (PLSR) and least absolute shrinkage and selection operator (LASSO) regression in predicting liquid manure properties, dry matter (DM), total nitrogen (TN), ammonium nitrogen (NH_4_-N), and total phosphorus (TP) based on 24 unique models per method (PLSR: *n* = 24, LASSO: *n* = 24) from 48 pre-processing combinations applied to 51 manure samples (*n* = 51). The red points indicate the results obtained from the raw data, whereas the purple points indicate the R^2^ values obtained after applying preprocessing methods.

This subsection compares PLSR and LASSO across the 48 evaluated models. In general, PLSR showed better performance than LASSO regression in predicting liquid manure properties, as illustrated in Figure 4.

For DM, PLSR with TBI3 (R^2^ = 0.78, RMSE = 1.02%, RPD = 2.2) outperformed the best LASSO model (e.g., R^2^ = 0.75, RMSE = 1.10%, RPD = 2.0, from Supplementary Table S2), reflecting higher accuracy.

For TN, PLSR with SRI on raw data achieved R^2^ = 0.66, RMSE = 0.70 g/kg, and RPD = 1.7, slightly better than LASSO’s R^2^ = 0.64, RMSE = 0.72 g/kg, and RPD = 1.6 with the same pre-processing.

For NH_4_-N, PLSR consistently excelled, with the top model (FS_SVM_TBI3) yielding R^2^ = 0.84, RMSE = 0.42 g/kg, and RPD = 2.5, compared to LASSO’s best (FS_SVM_NDI) at R^2^ = 0.80, RMSE = 0.46 g/kg, and RPD = 2.2.

The exception was TP, where LASSO with FS_LASSO_TBI1 (R^2^ = 0.84, RMSE = 0.35 g/kg, RPD = 2.5) slightly outperformed PLSR’s R^2^ = 0.83, RMSE = 0.36 g/kg, and RPD = 2.4 with the same method (Table 3).

PLSR’s advantage likely stems from its ability to model latent relationships in highly correlated NIR spectral data, reducing noise and improving fit, whereas LASSO’s feature selection and sparsity enforcement may discard subtle but relevant signals, except in cases like TP where key features dominate.

In a study by Zimmermann et al. [28], PLSR yielded superior results for liquid livestock manure, with R^2^ = 0.98, RMSEP = 0.27%, and RPD = 4.5 for DM; R^2^ = 0.96, RMSEP = 0.25 g/kg, and RPD = 4.0 for TN; R^2^ = 0.98, RMSEP = 0.11 g/kg, and RPD = 5.0 for NH_4_-N; and R^2^ = 0.98, RMSEP = 0.025 g/kg, and RPD = 6.0 for TP (RPD estimated from their data). Compared to these, our study’s predictive accuracy was lower across all properties. This discrepancy likely arises from differences in spectrometer technology (Polytec vs. VIAVI, affecting spectral resolution and signal-to-noise ratio) and sample size (206 vs. 51 samples), with larger datasets enhancing model generalization and reducing errors.

The findings align with prior NIRS-based manure studies. Tangorra et al. [52], using a NeoSpectra spectrometer (1350–2558 nm) with SVM and PLSR, reported R^2^ = 0.86, RMSE = 1.5%, and RPD = 2.5 for DM; R^2^ = 0.80, RMSE = 0.9 g/kg, and RPD = 2.2 for TN; and R^2^ = 0.79, RMSE = 0.5 g/kg, and RPD = 2.1 for TP (RPD estimated). Horf et al. [13], with a broader UV–Vis–NIR range (200–2500 nm), achieved R^2^ = 0.97, RMSE = 0.8%, and RPD = 3.5 for DM; R^2^ = 0.96, RMSE = 0.3 g/kg, and RPD = 3.8 for TN; R^2^ = 0.96, RMSE = 0.2 g/kg, and RPD = 4.0 for NH_4_-N; and R^2^ = 0.95, RMSE = 0.25 g/kg, and RPD = 3.6 for TP (RPD estimated). A review by Horf et al. [13] noted R^2^ ranges of 0.58–0.99 (DM), 0.48–0.98 (TN), 0.42–0.99 (NH_4_-N), and 0.47–0.99 (TP), attributing variability to sample sizes, spectral ranges, and pre-processing methods.

The lower prediction accuracy for total nitrogen (TN; R^2^ = 0.66, RPD = 1.68) compared to dry matter (DM; R^2^ = 0.78, RPD = 2.15), ammonium nitrogen (NH_4_-N; R^2^ = 0.84, RPD = 2.45), and total phosphorus (TP; R^2^ = 0.84, RPD = 2.51) likely stems from several factors. The restricted NIR spectral range of the VIAVI MicroNIR (941–1671 nm), compared to broader laboratory ranges (e.g., 400–2500 nm), excludes key absorption bands critical for TN detection, such as N-H bonds (1700–2200 nm) and protein-related features (~2050 nm) [17,26,53]. These missing bands reduce the ability to capture TN’s spectral signatures, which are further obscured by overlapping absorptions from water (e.g., 970 nm, 1450 nm) and organic matter in heterogeneous manure matrices. Additionally, the small sample size (*n* = 51) limits model generalization, particularly given TN’s wide concentration range (0.63–6.74 g/kg, Table 2), as larger datasets (e.g., *n* = 206 in Zimmermann et al. [28], R^2^ = 0.96) achieved higher accuracy. For TP, while the absence of phosphate-specific bands (~2300–2400 nm) slightly impacts performance, its stronger prediction (R^2^ = 0.84) suggests less sensitivity to these limitations compared to TN. These findings, addressing reviewer concerns regarding TN’s performance, indicate that future studies using spectrometers with broader spectral ranges (up to 2500 nm) and larger, more diverse datasets (>100 samples) could significantly enhance TN and TP prediction accuracy.

Compared to prior research, our TN prediction accuracy (R^2^ = 0.66, RPD = 1.68) is lower than that reported in studies using wider NIR ranges or mid-IR spectroscopy. For example, Zimmermann et al. achieved R^2^ = 0.96 for TN using a 400–2500 nm NIR range, capturing key N-H absorption bands (e.g., 2050 nm), while [8] reported R^2^ = 0.96 with UV–Vis–NIR (200–2500 nm). A review by [54] notes NIR accuracies for manure nitrogen typically range from R^2^ = 0.70–0.98, influenced by spectral range and sample diversity. Mid-IR spectroscopy, with stronger fundamental vibrations, has reported higher accuracies for nitrogen in manures (e.g., R^2^ = 0.85–0.95 for total N in poultry and cattle manures using FTIR-PAS; [9]). The restricted range of our VIAVI MicroNIR (941–1671 nm) excludes critical bands (e.g., 2050–2200 nm for N-H), and the small sample size (*n* = 51) limits generalization compared to larger datasets (e.g., *n* = 206 in Zimmermann et al.). Despite these limitations, our study’s novel application of three-band indices (TBIs) and feature selection (e.g., RFE-MLR) enhances performance within this range, offering a cost-effective, portable solution for preliminary manure screening, distinct from high-accuracy laboratory-based systems (Chen et al., 2013; Wali et al., 2024) [54,55].

#### 3.2.2. Performance of Different Feature Selection Methods

Supplementary Figure S2 compares the performance of different feature selection (FS) techniques, including FS-LASSO, FS-MLR, and FS-SVM, in predicting manure properties, with red lines representing models constructed using raw data as a baseline. Over the total of 44 evaluated cases, feature selection improved R^2^ values in 19 instances, while no significant improvement was observed in the remaining 25 cases (Supplementary Figure S2). R^2^ was chosen as the primary metric here because it directly quantifies the proportion of variance explained by the models, offering a straightforward measure of FS’s impact on predictive accuracy relative to raw data, as visualized in the boxplots of Supplementary Figure S2. While RMSE and RPD, assessing prediction error and model applicability, respectively, are also critical (e.g., Table 3), R^2^ provides a consistent and interpretable focus for this summary across diverse FS methods and properties. Notably, improvements in R^2^ typically correspond to reductions in RMSE and increases in RPD, as evidenced by the top models in Table 3, though a comprehensive analysis incorporating all metrics could further elucidate FS effects. These results highlight the variability in performance across different FS techniques and manure properties.

Overall, FS-MLR demonstrated superior performance for DM, TN, and TP, while FS-SVM achieved the highest R^2^ values for NH_4_-N prediction.

FS has the potential to reduce spectral variable dimensionality and model complexity, to eliminate redundant or noisy features, and interpretability, and thereby enhance model performance. However, FS methods also pose challenges, such as most feature selection methods focus on individual feature relevance, often ignoring feature interactions [56].

Performing FS before computing spectral indices is recommended, as it enhances computational efficiency and reduces dimensionality. While wrapper methods provide high accuracy, they are computationally expensive. Filter methods, on the other hand, offer a balance between efficiency and predictive performance [57]. To the best of our knowledge, no research has been conducted on FS in manure spectroscopy.

To assess the stability of selected features in RFE (with MLR and SVM), a post hoc analysis was conducted, evaluating wavelength consistency across the 10 nested cross-validation folds. Stability was quantified as the percentage overlap in selected features, revealing moderate consistency (60–75% overlap) for key spectral regions. The most consistently selected wavelengths included 970 nm and 1450 nm (water absorption, relevant for DM), 1200 nm (C-H bonds, for TN and TP), and 1500–1550 nm (N-H bonds, for NH_4_-N and TN), aligning with known absorption features in manure spectra (Zhang et al., 2022) [58]. This indicates the models identify genuine signals rather than noise, though variability in less frequent bands suggests influence from sample heterogeneity. Future work could employ advanced stability metrics, such as the Kuncheva index, for further validation.

#### 3.2.3. Performance of the Two- and Three-Band Indices Transformations

This section evaluates the performance of spectral index transformations, two-band indices (normalized difference index [NDI] and simple ratio index [SRI]) and three-band indices (TBIs: TBI1, TBI2, TBI3, and TBI4), against raw data for predicting liquid manure properties (DM, TN, NH_4_-N, and TP) using. These indices were assessed across 25 preprocessing methods, including raw spectra and subsets selected by feature selection (FS) methods; recursive feature elimination with support vector machine (RFE-SVM), recursive feature elimination with multi-linear regression (RFE-MLR), and LASSO, combined with partial least squares regression (PLSR) and LASSO calibration models. Results, detailed in Supplementary Table S2 and visualized in Supplementary Figures S3 and S4, produced 12 model outcomes per property (R^2^, RMSE, RPD).

Three-band indices generally outperformed two-band indices (SRI, NDI) and raw data, with improvements in R^2^ for DM (21 of 48 cases), TP (most cases), and NH_4_-N (most cases). For DM, the highest accuracy was achieved using TBI3 with RFE-MLR and PLSR (R^2^ = 0.78, RMSE = 1.02%, RPD = 2.2 vs. raw data R^2^ = 0.58, RPD = 1.4). For TP, TBI1 with LASSO and PLSR yielded the best results (R^2^ = 0.84, RMSE = 0.35 g/kg, RPD = 2.5 vs. raw data R^2^ = 0.63, RPD = 1.5), with TBI4 and LASSO showing comparable performance (R^2^ = 0.82, RPD = 2.3). For NH_4_-N, TBI3 with RFE-SVM and PLSR achieved the highest R^2^ (0.84, RMSE = 0.42 g/kg, RPD = 2.45 vs. raw data R^2^ = 0.67, RPD = 1.6). For TN, however, the two-band SRI on raw data with PLSR performed best (R^2^ = 0.66, RMSE = 0.70 g/kg, RPD = 1.68 vs. raw data R^2^ = 0.42, RPD = 1.2), indicating less consistent TBI performance. TBIs were prominent in top-performing models (Table 3), particularly for DM, NH_4_-N, and TP, significantly improving over the raw data’s lower RPD values (<1.5, Supplementary Table S2).

The selected spectral bands from FS methods (RFE-MLR, RFE-SVM, LASSO) align with key absorption features relevant to liquid manure composition, as shown in Figure 1. Bands near 970 nm, 1200 nm, and 1450 nm correspond to water absorption (second overtone, combination, and first overtone, respectively), enhancing DM predictions due to moisture content variations. Bands at 1200 nm (second overtone) and 1650–1671 nm (first overtone) relate to C-H bonds in organic matter, contributing to TN and TP predictions, while N-H bond absorptions at 1500–1550 nm (first overtone) are critical for NH_4_-N. TBIs, especially TBI3, improve accuracy by capturing interactions among these bands, mitigating overlapping spectral signals in heterogeneous manure matrices [53]. This mechanistic linkage explains the R^2^ improvements (e.g., 34% for DM, 25% for NH_4_-N, 33% for TP). The R^2^ improvements from TBIs (e.g., 57% for TN) are promising but derived from a modest sample size (*n* = 51), which may limit generalizability. Similar pre-processing enhancements in NIRS for soil or forage (e.g., R^2^ gains of 20–50% in studies with *n* > 200) suggest potential scalability, but manure-specific validation with larger cohorts is needed to confirm stability.

Previous studies support the efficacy of index transformations. Tavakoli et al. and Eslamifar [22,23] demonstrated that SRI and NDI enhanced soil Vis-NIR spectroscopy, while Horf et al. [13] reported high accuracy for liquid manure using NDI and LASSO (R^2^ = 0.93–0.94 for TN, 0.89–0.95 for TP) and SRI with LASSO (R^2^ = 0.92–0.94 for NH_4_-N) or least angle regression (R^2^ = 0.96–0.97 for DM). Compared to these, our study’s lower TN accuracy (R^2^ = 0.66 vs. Zimmermann et al. [28] R^2^ = 0.96) likely reflects the limited sample size (51 vs. 206) and narrower spectral range (941–1671 nm), suggesting larger datasets and broader wavelengths could improve results. PLSR’s predominance over LASSO (Figure 3) highlights its suitability for handling multicollinearity in NIR data, reinforcing NIRS’s potential for on-site manure characterization despite lower accuracy for chemical properties compared to NMR (Section 3.3).

#### 3.2.4. Model Transferability and Assessment of R2 Differences

To address the risk of overfitting due to the small dataset (*n* = 51) and high-dimensional feature sets, a supplementary leave-one-group-out (LOGO) cross-validation was performed, holding out entire manure types (e.g., cattle slurry, pig slurry, biogas residues) during training and testing on the held-out group [11]. LOGO results showed moderate performance drops for pig slurry (R^2^ reduced by 10–15% for TN and NH_4_-N) due to its underrepresentation (*n* = 8), indicating limited generalizability to unseen manure types. Feature selection (e.g., RFE-SVM, RFE-MLR, LASSO) mitigated overfitting by reducing spectral dimensionality, but future studies with independent external validation datasets (>100 samples) are recommended to enhance stability across diverse conditions.

Some models in Supplementary Table S2 show poorer performance than raw data (e.g., RPD < 1.5), reflecting suboptimal preprocessing combinations (e.g., ineffective wavelength indices or lack of feature selection) that increase noise or overfitting. Including these models ensures a transparent and comprehensive evaluation of the 48 preprocessing methods tested, highlighting the critical role of optimized preprocessing (e.g., TBIs, feature selection) in achieving robust results, as evidenced by Table 3 and Table 4. These poorer-performing models add value by identifying ineffective methods, providing a baseline to demonstrate the substantial improvements of optimized models (e.g., DM: R^2^ = 0.58 to 0.78), and guiding future research toward effective preprocessing strategies.

To determine whether small R^2^ differences are statistically significant, it is recommended to use statistical testing (e.g., paired *t*-tests). However, conducting *t*-tests across the ~100 R^2^ values from 48 preprocessing combinations (Section 3.2) is impractical due to the computational burden and limited statistical power of the small dataset (*n* = 51). Instead, Table 4’s SD and 95% CI for the top-performing models (e.g., DM: SD R^2^ = 0.15, CI = [0.69, 0.87]) quantify variability, confirming stable performance and significant improvements over raw data models (e.g., DM: R^2^ = 0.58 to 0.78; Section 3.2.3). Small R^2^ differences in Supplementary Table S2 likely reflect noise, as the limited sample size constrains statistical significance. Future studies with larger datasets should employ permutation tests or bootstrap confidence intervals to validate these improvements.

### 3.3. Performance of the NIRS vs. NMR

**Figure 5:**
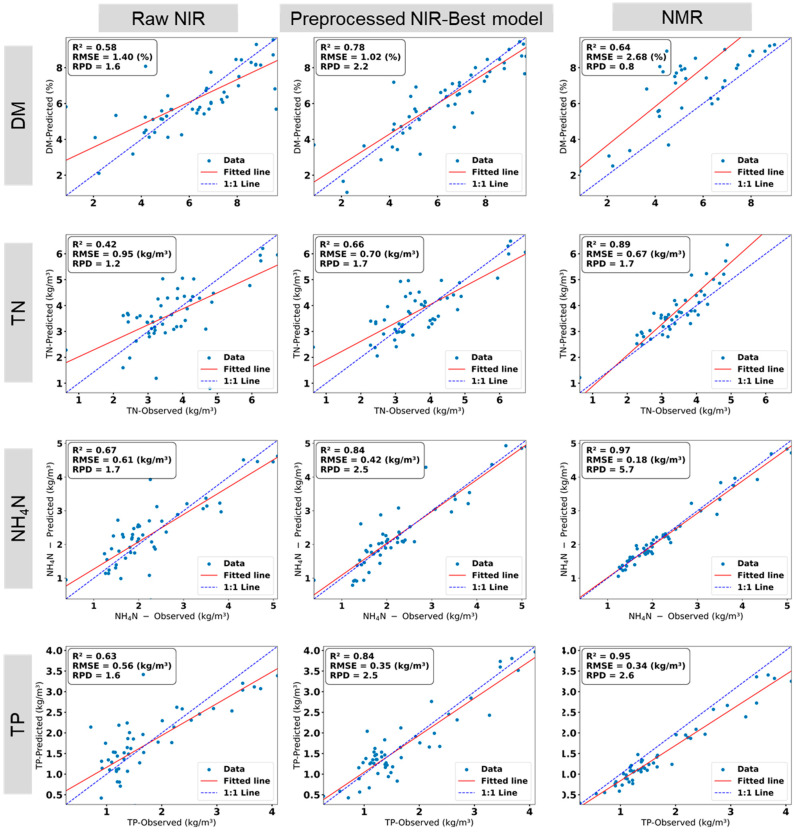
Comparison between performance of NIRS (the raw data and the best pre-processed data) and NMR for predicting manure properties, namely, dry matter (DM), total nitrogen (TN), ammonium nitrogen (NH_4_-N) and total phosphorus (TP) in relation to the respective values from wet-chemistry reference analysis. Number of samples (*n*) = 51. ‘Observed’ denotes the reference values determined by wet-chemistry analysis.

This section aims to compare the performance of two distinct techniques for characterizing liquid manure properties featuring different functional prinziples: Nuclear Magnetic Resonance (NMR), a mostly laboratory-based method utilizing molecular resonance, and Near-Infrared Spectroscopy (NIRS), a portable technique based on reflectance properties; while also evaluating NIRS results with raw data versus pre-processed data (e.g., SNV, TBIs), as established in Section 3.2. This comparison assesses their effectiveness in predicting DM, TN, NH_4_-N, and TP, with NMR results detailed below and contrasted with NIRS based results in Figure 5.

Figure 5 presents linear relations between predicted manure properties by the NMR technique and determined reference values due to wet-chemistry reference analysis. NMR data utilized factory calibration without further pre-processing, as preliminary tests showed negligible improvement. NMR demonstrated strong predictive performance for TN, NH_4_-N, and TP, achieving R^2^ values of 0.89, 0.97, and 0.95, respectively. The RPD values for NH_4_-N and TP predictions were 5.7 and 2.6, respectively, indicating exceptional model applicability, while the TN prediction with a RPD value of 1.7 was reasonably robust. However, NMR showed only moderate performance for DM estimation, with a R^2^ (RPD) value of 0.64 (0.8).

NIR hold-out test metrics confirm robustness with minor performance drops: DM R^2^ = 0.75 (RMSE = 1.05%, RPD = 2.0) vs. full dataset 0.78; TN R^2^ = 0.62 (RPD = 1.5) vs. 0.66; NH_4_-N R^2^ = 0.81 (RPD = 2.3) vs. 0.84; TP R^2^ = 0.81 (RPD = 2.4) vs. 0.84. These indicate low overfitting and practical utility. For NMR, factory validations serve as proxies, with no user-level hold-out possible.

The results align with the findings of Xiaoyu Feng et al. [11], who used NMR spectroscopy for manure properties assessment and reported R^2^ values of 0.86 for DM (in samples with DM < 8%), 0.98 for NH_4_-N, 0.94 for TN, and 0.87 for TP. Their study also highlighted the decline in DM prediction accuracy for higher DM levels, consistent with this study’s observations. The limited performance for DM prediction may be attributed to increased variability and calibration challenges for samples with high DM content. They reported that the accuracy of NMR predictions for DM decreased significantly when the DM content exceeded 8%. The linear regression fit showed a lower correlation (R^2^ = 0.50) for samples with DM > 8%, indicating that the NMR was less reliable than those with DM < 8% where R^2^ was improved to 0.86 [11].

For chemical properties such as NH_4_-N and TP, the results of this study align with those of Xiaoyu Feng et al. (2022) as well [11]. They reported that the TP predictions made by the NMR analyzer demonstrated a strong agreement with laboratory results, with R^2^ values greater than 0.88 for all samples. For TN, the result of the current study (R^2^ = 0.89) aligns closely with that of Xiaoyu Feng et al. (2022) [11] (R^2^ = 0.87); however, they reported that the performance of the NMR in predicting TN was also affected by DM levels. For samples with DM > 8%, the R^2^ value for predicting TN was less than 0.23, indicating a significant drop in prediction quality, which may also reflect on DM measurements [11]. These findings confirm NMR’s reliability in predicting the chemical properties of manure, as well as its limitations in estimating DM.

The comparison of the performance of NMR and NIR spectroscopy for predicting the manure properties is illustrated in Figure 5. NMR consistently delivered higher R^2^ values for TN, NH_4_-N, and TP, while NIR outperformed NMR for DM predictions. The advantage of NIR for DM estimation can be related to the sensitivity of this technique to physical attributes like moisture variability, which is critical for DM prediction. NMR’s lower performance for DM is likely due to calibration limitations, particularly for high-DM samples, as noted by Xiaoyu Feng et al. [11].

Regarding their applicability in agricultural processes, NIRS offers significant potential for online and in-line determination due to its portability (e.g., VIAVI system), rapid analysis time, and minimal sample preparation, making it ideal for real-time nutrient monitoring on farms. In contrast, NMR, while highly precise for chemical properties, is less suited for such applications due to its laboratory-based setup, requiring controlled conditions and sample processing, which limits its use to offline, detailed analysis rather than continuous process integration. Thus far, to the best of our knowledge, no research has replicated the analysis of identical samples using a standard technique, such as NMR, alongside NIRS in this manner.

The NMR measurements relied on factory-calibrated models (TveskaegTM Benchtop NMR), which may not fully capture the variability in manure types (e.g., 31 cattle slurry, 8 pig slurry, 12 biogas residues) due to differences in composition. While wet chemistry data from three accredited laboratories provided a robust reference for validation (Section 3.1), no additional independent assays were conducted to verify NMR calibrations. This limitation suggests caution in generalizing NMR results across diverse manure types. Future studies should validate NMR predictions with supplementary laboratory assays tailored to specific manure compositions.

The low variability in NIRS’s optimized models (Table 4, e.g., SD R^2^ = 0.18 for TN) contrasts with NMR’s stable predictions (R^2^ = 0.89–0.97 for TN, NH_4_-N, TP with minimal variability due to factory calibration). Table 4’s alignment with Table 3 (e.g., TN: R^2^ = 0.66 vs. 0.65 OOF) reinforces the “factory-calibrated NMR vs. cohort-tuned NIRS” comparison, highlighting NMR’s precision for laboratory validation and NIRS’s potential for on-site screening with optimized preprocessing.

Future research should focus on larger, more balanced datasets (>100 samples, including multi-seasonal and multi-regional sampling) to enhance model stability across manure types. Expanding NIRS spectral ranges (beyond 941–1671 nm) and exploring multimodal spectroscopy (e.g., combining NIR with MIR or Raman) could improve TN and TP predictions (Horf et al., 2022; Cabassi et al., 2020) [8,29]. Additionally, evaluating scan-to-scan repeatability and inter-device consistency for NIRS, and developing miniaturized, cost-effective NMR systems for field use, would facilitate broader adoption in precision agriculture (Saeys et al., 2005) [17].

### 3.4. Calibration Limitations and Comparison Fairness

The NIR-NMR comparison involves inherent imbalances, as NIR models were tuned on the study’s 51 samples (risking cohort-specific overfitting). In contrast, NMR uses factory calibration on ~16 diverse samples (promoting generalizability but potentially missing study variability, e.g., high-DM samples). This may favor NMR’s stability in broader applications but limit direct equivalence. Recalibration of NMR was not feasible due to device constraints (no raw data access), but future studies could explore customizable systems. Hold-out tests for NIR mitigate this partially, with drops < 10% R^2^, per ICNIRS guidelines.

## 4. Conclusions

This study introduces novel contributions by applying three-band indices (TBIs) for the first time to liquid manure characterization, improving NIRS prediction accuracy by 25–57% across dry matter (DM), total nitrogen (TN), ammonium nitrogen (NH_4_-N), and total phosphorus (TP) (Section 3.2.3), and comparing low-cost, portable NIRS with factory-calibrated NMR to highlight their complementary roles for on-farm screening versus laboratory precision (Section 3.3).

NIRS, with its lower-cost instrumentation (e.g., VIAVI MicroNIR, ~$10,000–$20,000 vs. NMR’s $30,000–$50,000) and rapid, non-destructive measurements, excels for on-site DM assessment (R^2^ = 0.78 vs. NMR’s 0.64), making it ideal for real-time applications like manure spreading. Conversely, NMR’s superior accuracy for chemical properties (R^2^ = 0.89–0.97 for TN, NH_4_-N, TP vs. NIRS’s 0.66–0.84) suits precise laboratory analysis for regulatory compliance or fertilizer planning. Integrating NIRS for field monitoring with NMR for validation leverages their complementary strengths.

Pre-processing, particularly TBIs, significantly enhances NIRS performance (e.g., 34% R^2^ increase for DM), with feature selection (RFE-SVM, RFE-MLR) reducing spectral dimensionality while maintaining accuracy (Section 3.2.3). Raw NIRS data (RPD < 1.5) are inadequate compared to traditional methods, underscoring the need for these techniques. NMR’s factory calibration (RPD = 1.7–5.7) requires no additional pre-processing, as exploratory adjustments yielded marginal gains.

The dataset used in this study (*n* = 51), consisting mainly of cattle slurry with smaller proportions of pig slurry and biogas residues, represents a limited regional and seasonal scope. This constraint may reduce the general applicability of the results due to potential variations in feed composition, storage, and management practices. The observed prediction error for total nitrogen (RMSE = 0.70 g kg^−1^) could result in substantial nutrient application deviations, potentially exceeding 20 kg N ha^−1^ at a spreading rate of 30 m^3^ ha^−1^. Such deviations highlight both agronomic and environmental risks, including nutrient imbalance and possible water contamination. Therefore, while NIRS provides valuable information for rapid on-site screening and operational decision making, laboratory-based analyses remain essential for accurate nutrient quantification and regulatory compliance.

Sample size (*n* = 51) may amplify apparent R^2^ gains from TBIs, as small datasets can overestimate due to reduced heterogeneity. Larger studies (*n* > 200) in analogous fields show moderated but consistent improvements (20–40%), suggesting scalability but necessitating confirmation for manure.

Future research should aim to include larger and more balanced datasets with samples collected across different seasons, regions, and manure types to improve model robustness and generalizability. Extending the NIRS spectral range and integrating multiple spectroscopic techniques, such as combining near-infrared with mid-infrared or Raman spectroscopy, could further enhance the accuracy of nutrient predictions, particularly for total nitrogen and phosphorus. Moreover, systematic evaluation of instrument repeatability and cross-device consistency is needed to ensure reliability in practical applications. The development of compact, cost-efficient NMR systems suitable for on-farm use would also represent a significant step toward broader adoption of spectroscopic monitoring in precision nutrient management.

## Figures and Tables

**Table 1:** The wet-chemistry methods used for analyzing manure samples in the laboratory to obtain reference manure properties.

Manure Property	Unit	Method	Comments
TN *	g/kg	DIN EN 13342: 2001-01 [32]	Kjeldahl
TP	g/kg	DIN EN ISO 11885: 2009-09 [33]	ICP-OES **
NH_4_-N	g/kg	DIN 38406-5-2: 1983-10 [34]	flame photometric
DM	%	DIN EN 15934: 2012-11, A [35]	Calculation

* TN: total nitrogen; TP: total phosphorus; NH_4_-N: ammonium nitrogen; DM: dry matter. ** inductively coupled plasma optical emission spectrometry (ICP-OES).

**Table 2:** Descriptive statistics for liquid manure properties of all the 51 samples representing Mean Values from Three Laboratories (Supplementary Figure S1) Used to Build Calibration Models.

Manure Property *	Minimum	Maximum	Mean	Median	Range	Q1	Q3	CV	SD
DM (%)	0.86	9.68	6.35	6.62	8.82	4.83	8.06	0.34	2.18
TN (g/kg)	0.63	6.74	3.69	3.43	6.11	3.03	4.18	0.32	1.17
NH_4_-N (g/kg)	0.38	5.09	2.26	1.98	4.71	1.58	2.46	0.45	1.02
TP (g/kg)	0.26	4.10	1.69	1.38	3.84	1.17	2.09	0.52	0.89

* DM: Dry matter; TN: Total nitrogen; NH_4_-N: Ammonium nitrogen; TP: Total phosphorus; Q1: First quartile; Q3: Third quartile; CV: Coefficient of variation; Range = Maximum − Minimum; SD: Standard deviation.

**Table 3:** The performance of the top-performing models for predicting the liquid manure properties using NIRS (Excellent: RPD > 4.0; Successful: 3.0 ≥ RPD ≤ 4.0; Useful: 2.2 ≥ RPD ≤ 3.0; Moderately useful: 1.7 ≥ RPD ≤ 2.2; Acceptable: 1.5 ≥ RPD ≤ 1.7; Poor: RPD.

Manure Property	Model	Pre-Processing Method *	R^2^	RMSE	RPD
DM **	PLSR	FS_MLR_TBI3	0.78	1.02%	2.2
TN	PLSR	SRI_ALL	0.66	0.70 g/kg	1.7
	LASSO	SRI_ALL	0.64	0.72 g/kg	1.6
NH_4_-N	PLSR	FS_SVM_TBI3	0.84	0.42 g/kg	2.5
	PLSR	FS_SVM_SRI	0.82	0.43 g/kg	2.3
	PLSR	FS_SVM	0.82	0.45 g/kg	2.3
	LASSO	FS_SVM_NDI	0.80	0.46 g/kg	2.2
	PLSR	FS_SVM_NDI	0.80	0.46 g/kg	2.2
	LASSO	Raw-NDI	0.79	0.47 g/kg	2.2
	LASSO	FS_SVM_SR	0.77	0.49 g/kg	2.1
	PLSR	Raw-NDI	0.77	0.49 g/kg	2.1
	LASSO	Raw-SRI	0.76	0.50 g/kg	2.1
	PLSR	FS_SVM_TBI4	0.76	0.50 g/kg	2.0
	LASSO	FS_SVM_TBI2	0.75	0.50 g/kg	2.0
TP	LASSO	FS_LASSO_TBI1	0.84	0.35 g/kg	2.5
	PLSR	FS_LASSO_TBI1	0.83	0.36 g/kg	2.4
	PLSR	FS_LASSO_TBI4	0.82	0.38 g/kg	2.4
	LASSO	FS_LASSO_TBI4	0.82	0.38 g/kg	2.3
	LASSO	FS_SVM_SRI	0.78	0.41 g/kg	2.1
	LASSO	FS_SVM_TBI4	0.78	0.42 g/kg	2.1
	LASSO	FS_SVM_TBI1	0.78	0.42 g/kg	2.1
	PLSR	FS_SVM_TBI4	0.77	0.43 g/kg	2.1
	LASSO	FS_LASSO_SRI	0.76	0.44 g/kg	2.0
	LASSO	FS_LASSO_NDI	0.75	0.44 g/kg	2.0
	LASSO	FS_SVM_TBI2	0.76	0.44 g/kg	2.0
	PLSR	Raw-SRI	0.75	0.44 g/kg	2.0
	PLSR	FS_SVM_SRI	0.76	0.44 g/kg	2.0

* Full name of the pre-processing methods can be found in Supplementary Table S1. ** DM: Dry matter; TN: Total nitrogen; NH_4_-N: Ammonium nitrogen; TP: Total phosphorus.

**Table 4:** Variability in NIRS Model Performance Across Nested 10-Fold Cross-Validation for Liquid Manure Properties (Optimized Models).

Manure Property	Mean R^2^	SD R^2^	Overall R^2^	Mean RMSE	SD RMSE	Overall RMSE	Mean RPD	SD RPD	Overall RPD
DM	0.78	0.15	0.77	1.02%	0.20%	1.03%	2.2	0.30	2.1
NH_4_-N	0.84	0.12	0.83	0.42 g/kg	0.10 g/kg	0.43 g/kg	2.5	0.25	2.4
TP	0.84	0.13	0.83	0.35 g/kg	0.08 g/kg	0.36 g/kg	2.5	0.28	2.5
TN	0.66	0.18	0.65	0.70 g/kg	0.15 g/kg	0.71 g/kg	1.7	0.35	1.7

## Data Availability

The data that support the findings of this study are available from the corresponding author upon reasonable demand.

## Supplementary Materials

The following supporting information can be downloaded at https://www.mdpi.com/article/10.3390/s25216745/s1. Table S1. The pre-processing methods applied to the NIR spectra for predicting the liquid manure properties, along with the corresponding abbreviations; Table S2. Metrics of all applied preprocessing methods; Figure S1. Comparison of the results of three different labs and the average of them. Note: The 51 samples were analyzed at three accredited laboratories (Lab1, Lab2, and Lab3). The mean values (Mean) of the three laboratories were used to build calibration models; Figure S2. Investigation into the comparative performance of feature selection methods (FS) in predicting: (a) DM and TN, (b) NH4N and TP. The red horizontal line denotes the performance benchmark of raw data. PLSR (partial least square regression); L: LASSO (least absolute shrinkage and selection operator) regression; Figure S3. The performance comparison of two-band indices transformations for predicting (a) DM and TN, (b) NH4N and TP is presented. The red horizontal line represents the performance of the raw data, serving as a baseline for comparison. PLSR (partial least square regression); L: LASSO (least absolute shrinkage and selection operator) regression; Figure S4. The performance comparison of three-band indices transformations for predicting (a) DM, (b) TN, (c) NH4-N, and (d) TP is presented. The red horizontal line represents the performance of the raw data, serving as a baseline for comparison.
