# Supplementary material for: Low-Cost NIR Spectroscopy Versus NMR Spectroscopy for Liquid Manure Characterization
Source: Sensors (Basel). 2025 Nov 4;25(21):6745. doi: 10.3390/s25216745 (PMC12609543; doi:10.3390/s25216745)
Supplement: Supplementary file 1 [file sensors-25-06745-s001.zip › sensors-3841366-supplementary.pdf]

Supplementary Table S1. The pre-processing methods applied to the NIR spectra for predicting the liquid manure properties, along with the corresponding abbreviations.

**Pre-processing method**	**Abbreviation for the pre-processing methods**	**Abbreviation for the models with PLSR**	**Abbreviation for the models with LASSO**
Smoothed and downsampled raw data (5 nm resolution)	Raw	P_Raw	L_Raw
Pre-processed Raw data with SNV	Raw-SNV	P_Raw-SNV	L_Raw-SNV
NDI transformation on Raw data	Raw-NDI	P_Raw-NDI	L_Raw-NDI
SRI transformation on Raw data	Raw-SRI	P_Raw-SRI	L_Raw-SRI
Feature selection using LASSO	FS-LASSO	P_FS-LASSO	L _FS-LASSO
Feature selection using RFE-MLR	FS-MLR	P_FS-MLR	L _FS-MLR
Feature selection using RFE-SVM	FS-SVM	P_FS-SVM	L _FS-SVM
NDI transformation on features selected by LASSO	FS-LASSO-NDI	P_FS-LASSO-NDI	L _FS-LASSO-NDI
NDI transformation on features selected by RFE-MLR	FS-MLR-NDI	P_FS-MLR-NDI	L _FS-MLR-NDI
NDI transformation on features selected by RFE-SVM	FS-SVM-NDI	P_FS-SVM-NDI	L _FS-SVM-NDI
SRI transformation on features selected by LASSO	FS-LASSO-SRI	P_FS-LASSO-SRI	L _FS-LASSO-SRI
SRI transformation on features selected by RFE-MLR	FS-MLR-SRI	P_FS-MLR-SRI	L _FS-MLR-SRI
SRI transformation on features selected by RFE-SVM	FS-SVM-SRI	P_FS-SVM-SRI	L _FS-SVM-SRI
TBI1 transformation on features selected by LASSO	FS-LASSO-TBI1	P_FS-LASSO-TBI1	L _FS-LASSO-TBI1
TBI1 transformation on features selected by RFE-MLR	FS-MLR-TBI1	P_FS-MLR-TBI1	L _FS-MLR-TBI1
TBI1 transformation on features selected by RFE-SVM	FS-SVM-TBI1	P_FS-SVM-TBI1	L _FS-SVM-TBI1
TBI2 transformation on features selected by LASSO	FS-LASSO-TBI2	P_FS-LASSO-TBI2	L _FS-LASSO-TBI2
TBI2 transformation on features selected by RFE-MLR	FS-MLR-TBI2	P_FS-MLR-TBI2	L _FS-MLR-TBI2

TBI2 transformation on features selected by RFE-SVM	FS-SVM-TBI2	P_FS-SVM-TBI2	L _FS-SVM-TBI2
TBI3 transformation on features selected by LASSO	FS-LASSO-TBI3	P_FS-LASSO-TBI3	L _FS-LASSO-TBI3
TBI3 transformation on features selected by RFE-MLR	FS-MLR-TBI3	P_FS-MLR-TBI3	L _FS-MLR-TBI3
TBI3 transformation on features selected by RFE-SVM	FS-SVM-TBI3	P_FS-SVM-TBI3	L _FS-SVM-TBI3
TBI4 transformation on features selected by LASSO	FS-LASSO-TBI4	P_FS-LASSO-TBI4	L _FS-LASSO-TBI4
TBI4 transformation on features selected by RFE-MLR	FS-MLR-TBI4	P_FS-MLR-TBI4	L _FS-MLR-TBI4
TBI4 transformation on features selected by RFE-SVM	FS-SVM-TBI4	P_FS-SVM-TBI4	L _FS-SVM-TBI4

* P: PLSR (partial least square regression); L: LASSO (least absolute shrinkage and selection operator) regression; FS: feature selection; SNV: standard normal variate; NDI: normalized difference indices; SRI: simple ratio indices; TBI: three-band indices; RFE: recursive feature elimination; SVM: support vector machine; MLR: multi-linear regression.

To make the abbreviations understandable, please follow the structure below:
Regressor: PLSR(P)/LASSO(L); Feature selection methods: LASSO(FS_LASSO)/SVM(FS_SVM)/MLR(FS_MLR); Preprocessing: SNV/NDI/SRI/TBI

Supplementary Table S2. Metrics of all applied preprocessing methods.

DM	R^2	RMSE (%)	RPD
P_Raw	0.58	1.40	1.56
L_Raw	0.57	1.42	1.53
P_Raw_SNV	0.57	1.42	1.53
L_Raw_SNV	0.58	1.40	1.56
L_FS-LASSO	0.62	1.34	1.63
L_FS_MLR	0.58	1.42	1.54
L_FS_SVM			
P_FS-LASSO	0.65	1.30	1.68
P_FS_MLR	0.62	1.35	1.62
P_FS_SVM			
P_Raw-SRI	0.73	1.19	1.84
L_Raw-NDI	0.60	1.37	1.59
P_Raw-NDI	0.67	1.28	1.71
L_Raw-SRI	0.65	1.29	1.69
L_FS_LASSO_SRI	0.67	1.25	1.75
L_FS_LASSO_NDI	0.65	1.27	1.71
L_FS_MLR_NDI	0.67	1.25	1.75
L_FS_MLR_SRI	0.67	1.26	1.74
L_FS_SVM_NDI			
L_FS_SVM_SRI			
P_FS_LASSO_SRI	0.65	1.28	1.70
P_FS_LASSO_NDI	0.50	1.53	1.43
P_FS_MLR_NDI	0.51	1.56	1.40
P_FS_MLR_SRI	0.78	1.19	1.84
P_FS_SVM_NDI			
P_FS_SVM_SRI			
L_FS_LASSO_TBI1	0.65	1.29	1.70
L_FS_LASSO_TBI2	0.60	1.37	1.59
L_FS_LASSO_TBI3	0.68	1.25	1.74
L_FS_LASSO_TBI4	0.69	1.21	1.80
P_FS_LASSO_TBI1	0.70	1.19	1.83
P_FS_LASSO_TBI2	0.65	1.29	1.70
P_FS_LASSO_TBI3	0.74	1.12	1.95
P_FS_LASSO_TBI4	0.67	1.24	1.76
L_FS_MLR_TBI1	0.71	1.17	1.86
L_FS_MLR_TBI2	0.67	1.24	1.76
L_FS_MLR_TBI3	0.62	1.33	1.64
L_FS_MLR_TBI4	0.69	1.22	1.79
P_FS_MLR_TBI1	0.64	1.32	1.66
P_FS_MLR_TBI2	0.74	1.11	1.96
P_FS_MLR_TBI3	0.78	1.02	2.15
P_FS_MLR_TBI4	0.63	1.33	1.64

TN	R^2	RMSE (g/kg)	RPD
P_Raw	0.42	0.95	1.23
L_Raw	0.28	0.99	1.18
P_Raw_SNV	0.27	1.01	1.16
L_Raw_SNV	0.51	0.84	1.40
L_FS-LASSO	0.21	1.04	1.13
L_FS_MLR	0.32	0.97	1.21
L_FS_SVM	N/S	N/S	N/S
P_FS-LASSO	0.22	1.04	1.13

P_FS_MLR	0.55	1.62	0.72
P_FS_SVM	N/S	N/S	N/S
P_Raw-SRI	0.66	0.70	1.68
L_Raw-NDI	0.57	0.77	1.52
P_Raw-NDI	0.56	0.82	1.44
L_Raw-SRI	0.64	0.72	1.63
L_FS_LASSO_SRI	0.17	1.06	1.10
L_FS_LASSO_NDI	0.17	1.06	1.11
L_FS_MLR_NDI	0.45	0.88	1.33
L_FS_MLR_SRI	0.37	0.96	1.22
L_FS_SVM_NDI	N/S	N/S	N/S
L_FS_SVM_SRI	N/S	N/S	N/S
P_FS_LASSO_SRI	0.28	1.71	0.69
P_FS_LASSO_NDI	0.31	1.74	0.67
P_FS_MLR_NDI	0.24	1.76	0.67
P_FS_MLR_SRI	0.49	1.63	0.72
P_FS_SVM_NDI	N/S	N/S	N/S
P_FS_SVM_SRI	N/S	N/S	N/S
L_FS_LASSO_TBI1	0.22	1.02	1.14
L_FS_LASSO_TBI2	0.19	1.05	1.12
L_FS_LASSO_TBI3	0.17	1.06	1.10
L_FS_LASSO_TBI4	0.20	1.04	1.12
P_FS_LASSO_TBI1	0.22	1.04	1.13
P_FS_LASSO_TBI2	0.24	1.02	1.15
P_FS_LASSO_TBI3	0.21	1.04	1.13
P_FS_LASSO_TBI4	0.22	1.04	1.13
L_FS_MLR_TBI1	0.34	1.69	0.69
L_FS_MLR_TBI2	0.46	1.60	0.73
L_FS_MLR_TBI3	0.18	1.77	0.66
L_FS_MLR_TBI4	0.43	1.67	0.70
P_FS_MLR_TBI1	0.51	1.70	0.69
P_FS_MLR_TBI2	0.47	1.63	0.72
P_FS_MLR_TBI3	0.37	1.71	0.69
P_FS_MLR_TBI4	0.49	1.71	0.69

**NH_4N**	**R^2**	**RMSE (g/kg)**	**RPD**
P_Raw	0.67	0.61	1.67
L_Raw	0.46	0.76	1.34
P_Raw_SNV	0.49	0.72	1.41
L_Raw_SNV	0.58	0.67	1.53
L_FS-LASSO	0.23	0.88	1.15
L_FS_MLR	0.25	0.88	1.16
L_FS_SVM	0.54	0.70	1.46
P_FS-LASSO	0.23	0.89	1.14
P_FS_MLR	0.33	1.64	0.62
P_FS_SVM	0.82	0.45	2.26
P_Raw-SRI	0.76	0.52	1.96
L_Raw-NDI	0.79	0.47	2.15
P_Raw-NDI	0.77	0.49	2.06
L_Raw-SRI	0.76	0.50	2.05
L_FS_LASSO_SRI	0.15	0.93	1.10
L_FS_LASSO_NDI	0.15	0.93	1.10

L_FS_MLR_NDI	0.62	0.64	1.60
L_FS_MLR_SRI	0.60	0.65	1.56
L_FS_SVM_NDI	0.80	0.46	2.22
L_FS_SVM_SRI	0.77	0.49	2.09
P_FS_LASSO_SRI	0.13	1.73	0.59
P_FS_LASSO_NDI	0.22	1.69	0.60
P_FS_MLR_NDI	0.36	1.67	0.61
P_FS_MLR_SRI	0.57	1.57	0.65
P_FS_SVM_NDI	0.80	0.46	2.20
P_FS_SVM_SRI	0.82	0.43	2.34
L_FS_LASSO_TBI1	0.31	0.86	1.18
L_FS_LASSO_TBI2	0.25	0.90	1.13
L_FS_LASSO_TBI3	0.31	0.85	1.19
L_FS_LASSO_TBI4	0.24	0.92	1.11
P_FS_LASSO_TBI1	0.33	0.86	1.19
P_FS_LASSO_TBI2	0.34	0.85	1.20
P_FS_LASSO_TBI3	0.34	0.85	1.20
P_FS_LASSO_TBI4	0.27	0.90	1.13
L_FS_MLR_TBI1	0.24	1.70	0.60
L_FS_MLR_TBI2	0.21	1.69	0.60
L_FS_MLR_TBI3	0.34	1.66	0.61
L_FS_MLR_TBI4	0.29	1.69	0.60
P_FS_MLR_TBI1	0.31	1.78	0.57
P_FS_MLR_TBI2	0.54	1.56	0.65
P_FS_MLR_TBI3	0.49	1.63	0.63
P_FS_MLR_TBI4	0.32	1.74	0.58
L_FS_SVM_TBI1	0.63	0.64	1.59
L_FS_SVM_TBI2	0.75	0.50	2.03
L_FS_SVM_TBI3	0.66	0.62	1.64
L_FS_SVM_TBI4	0.62	0.64	1.58
P_FS_SVM_TBI1	0.75	0.51	1.98
P_FS_SVM_TBI2	0.68	0.60	1.71
P_FS_SVM_TBI3	0.84	0.42	2.45
P_FS_SVM_TBI4	0.76	0.50	2.03

**TP**	**R^2**	**RMSE (g/kg)**	**RPD**
P_Raw	0.63	0.56	1.60
L_Raw	0.58	0.57	1.56
P_Raw_SNV	0.55	0.60	1.47
L_Raw_SNV	0.63	0.56	1.58
P_FS_SVM	0.65	0.54	1.64
L_FS_SVM	0.60	0.55	1.60
P_FS_MLR	0.71	0.50	1.76
L_FS_MLR	0.45	0.65	1.36
P_FS-LASSO	0.65	0.54	1.64
L_FS-LASSO	0.60	0.56	1.59
P_Raw-SRI	0.75	0.44	2.00
P_Raw-NDI	0.70	0.50	1.79
L_Raw-NDI	0.72	0.48	1.85

L_Raw-SRI	0.75	0.45	1.99
P_FS_SVM_SRI	0.76	0.44	2.00
P_FS_SVM_NDI	0.74	0.47	1.90
P_FS_MLR_SRI	0.66	0.53	1.68
P_FS_MLR_NDI	0.18	0.80	1.10
P_FS_LASSO_SRI	0.73	0.46	1.93
P_FS_LASSO_NDI	0.52	0.62	1.44
L_FS_SVM_SRI	0.78	0.41	2.14
L_FS_SVM_NDI	0.75	0.45	1.97
L_FS_MLR_SRI	0.55	0.59	1.51
L_FS_MLR_NDI	0.55	0.59	1.51
L_FS_LASSO_SRI	0.76	0.44	2.04
L_FS_LASSO_NDI	0.75	0.44	2.02
P_FS_SVM_TBI4	0.77	0.43	2.06
P_FS_SVM_TBI3	0.72	0.47	1.90
P_FS_SVM_TBI2	0.73	0.47	1.90
P_FS_SVM_TBI1	0.69	0.50	1.77
P_FS_MLR_TBI4	0.68	0.50	1.76
P_FS_MLR_TBI3	0.62	0.57	1.56
P_FS_MLR_TBI2	0.66	0.53	1.67
P_FS_MLR_TBI1	0.69	0.49	1.80
P_FS_LASSO_TBI4	0.82	0.38	2.35
P_FS_LASSO_TBI3	0.73	0.47	1.89
P_FS_LASSO_TBI2	0.71	0.48	1.85
P_FS_LASSO_TBI1	0.83	0.36	2.44
L_FS_SVM_TBI4	0.78	0.42	2.11
L_FS_SVM_TBI3	0.70	0.49	1.81
L_FS_SVM_TBI2	0.76	0.44	2.00
L_FS_SVM_TBI1	0.78	0.42	2.11
L_FS_MLR_TBI4	0.62	0.55	1.63
L_FS_MLR_TBI3	0.54	0.61	1.46
L_FS_MLR_TBI2	0.55	0.59	1.50
L_FS_MLR_TBI1	0.59	0.57	1.57
L_FS_LASSO_TBI4	0.82	0.38	2.34
L_FS_LASSO_TBI3	0.74	0.46	1.95
L_FS_LASSO_TBI2	0.72	0.46	1.91
L_FS_LASSO_TBI1	0.84	0.35	2.51

P: PLSR (partial least square regression); L: LASSO (least absolute shrinkage and selection operator) regression; FS: feature selection; SNV: standard normal variate; NDI: normalized difference indices; SRI: simple ratio indices; TBI: two-band indices; RFE: recursive feature elimination; SVM: support vector machine; MLR: multi-linear regression.

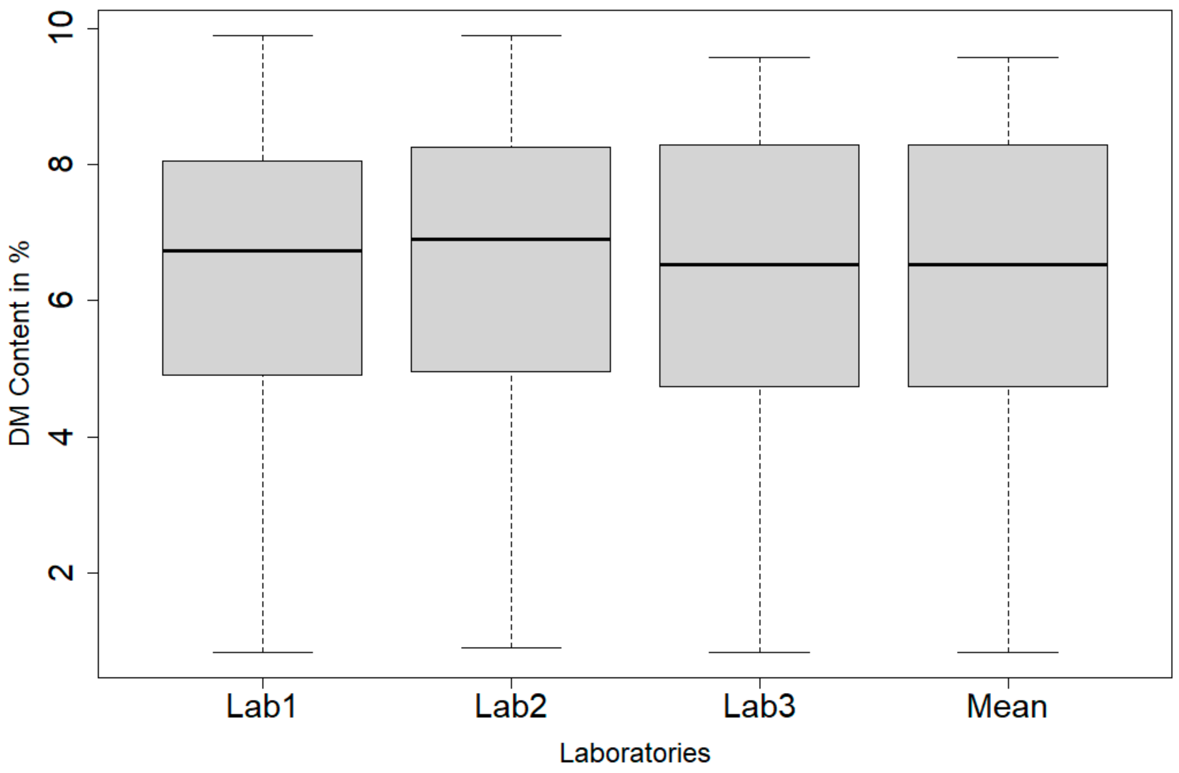

10
8
6
4
2
DM Content in %
Lab1
Lab2
Lab3
Mean
Laboratories

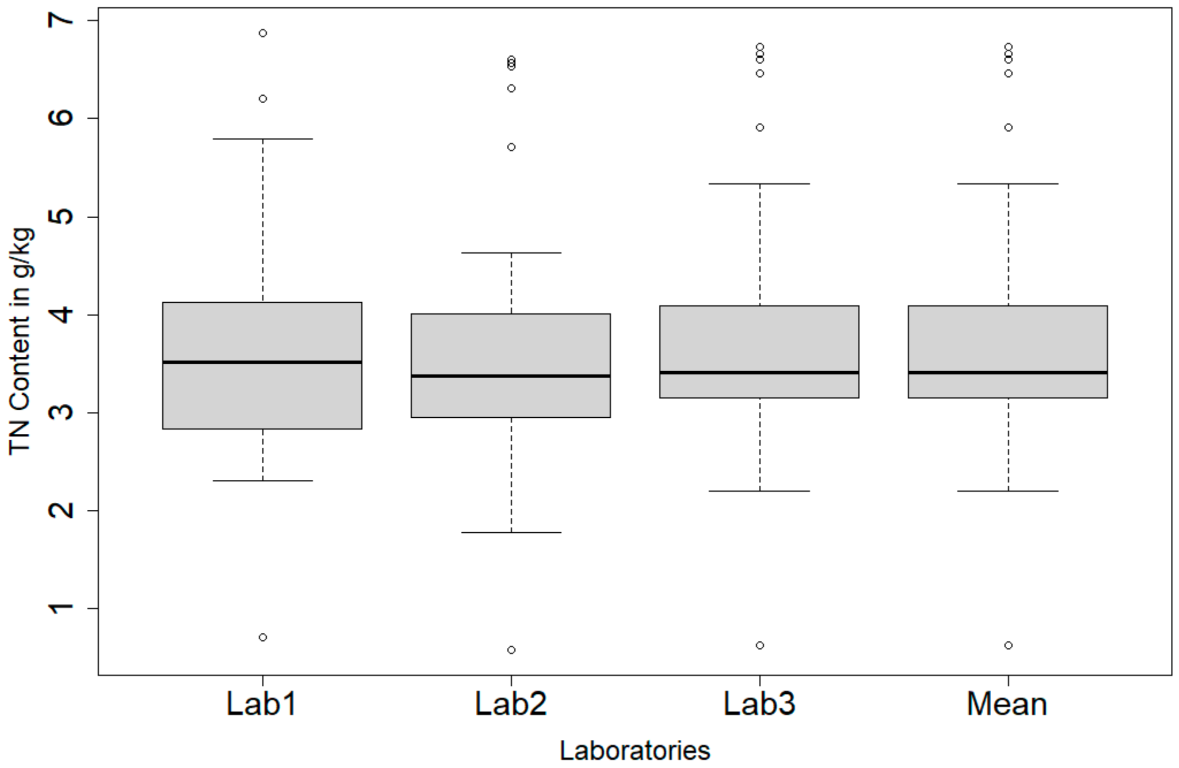

7
6
5
4
3
2
1
TN Content in g/kg
Lab1
Lab2
Lab3
Mean
Laboratories

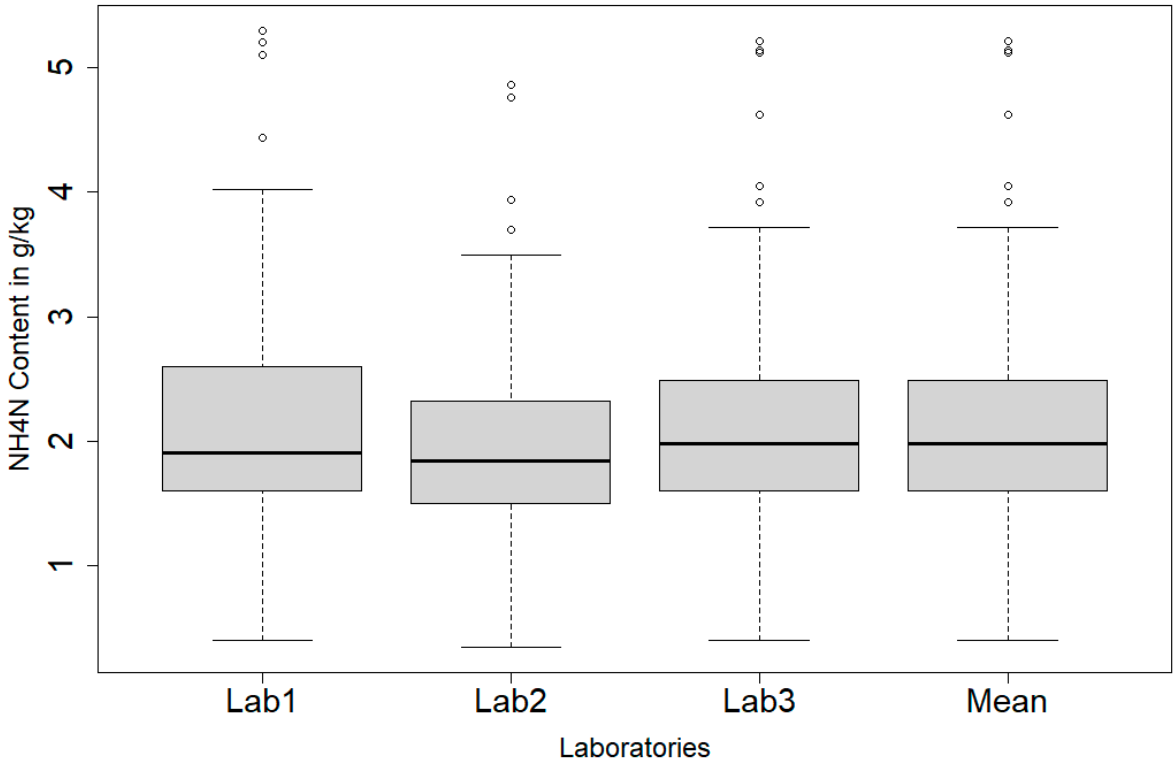
NH4N Content in g/kg
1
2
3
4
5
Lab1
Lab2
Lab3
Mean
Laboratories

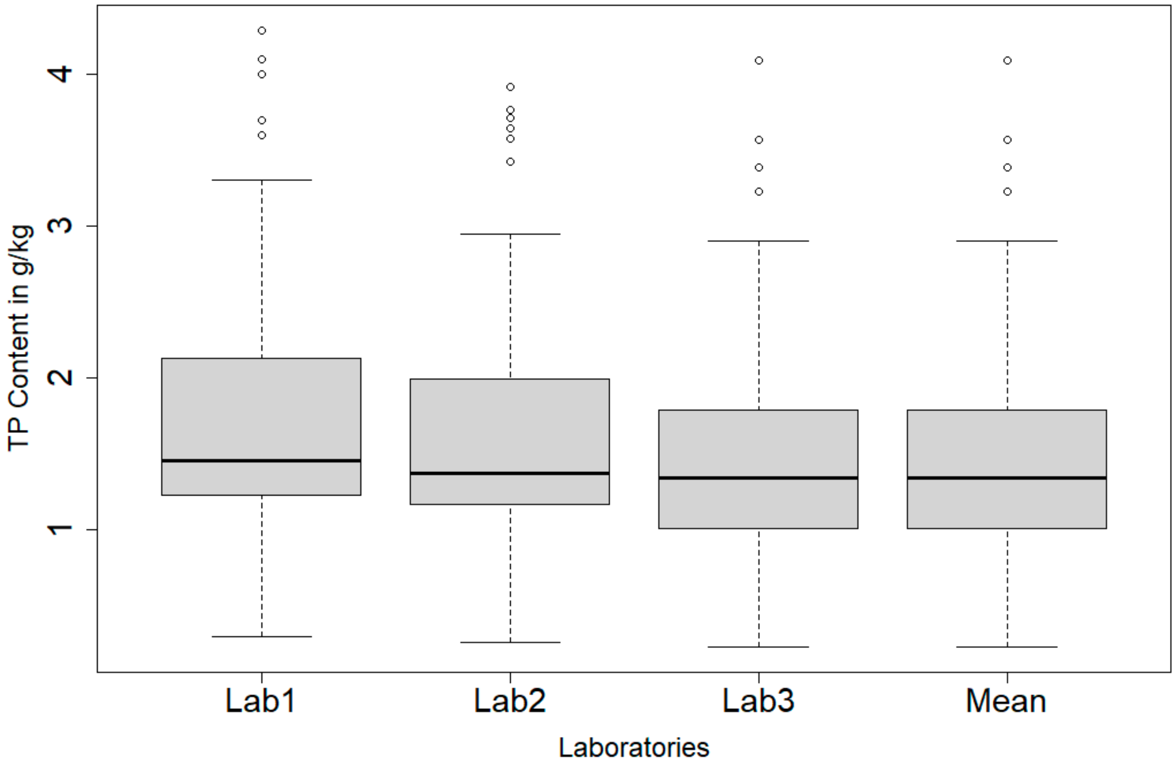
TP Content in g/kg
1
2
3
4
Lab1
Lab2
Lab3
Mean
Laboratories

Supplementary Figure S1. Comparison of the results of three different labs and the average of them. Note: The 51 samples were analyzed at three accredited laboratories (Lab1, Lab2, and Lab3). The mean values (Mean) of the three laboratories were used to build calibration models.

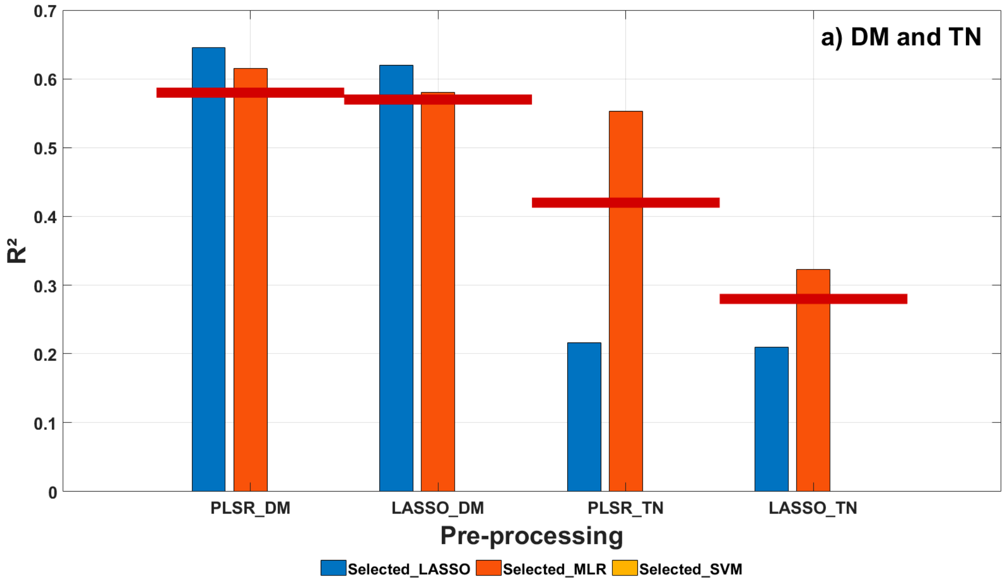

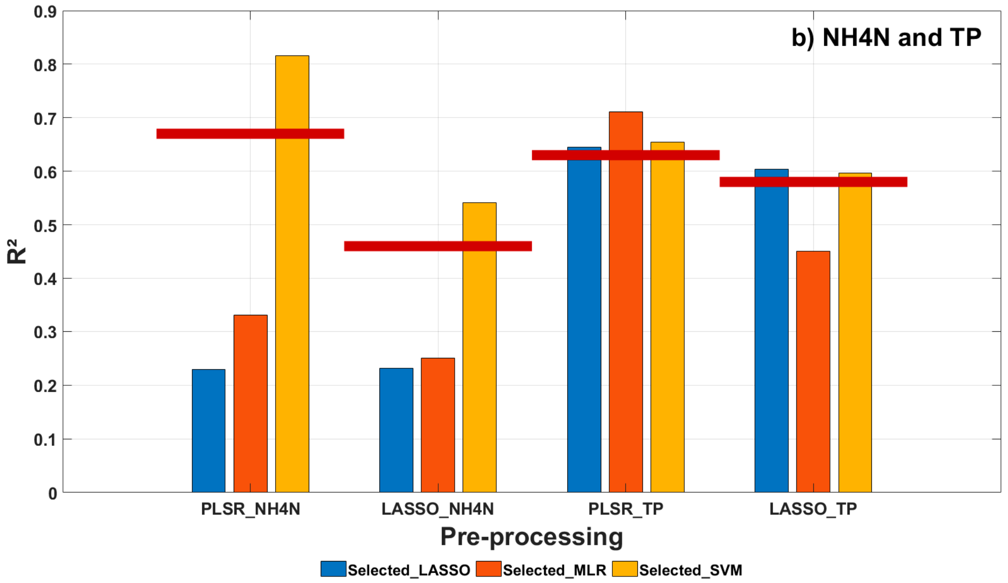

Supplementary Figure S2. Investigation into the comparative performance of feature selection methods (FS) in predicting: a) DM and TN, b) NH4N and TP. The red horizontal line denotes the performance benchmark of raw data. PLSR (partial least square regression); L: LASSO (least absolute shrinkage and selection operator) regression.

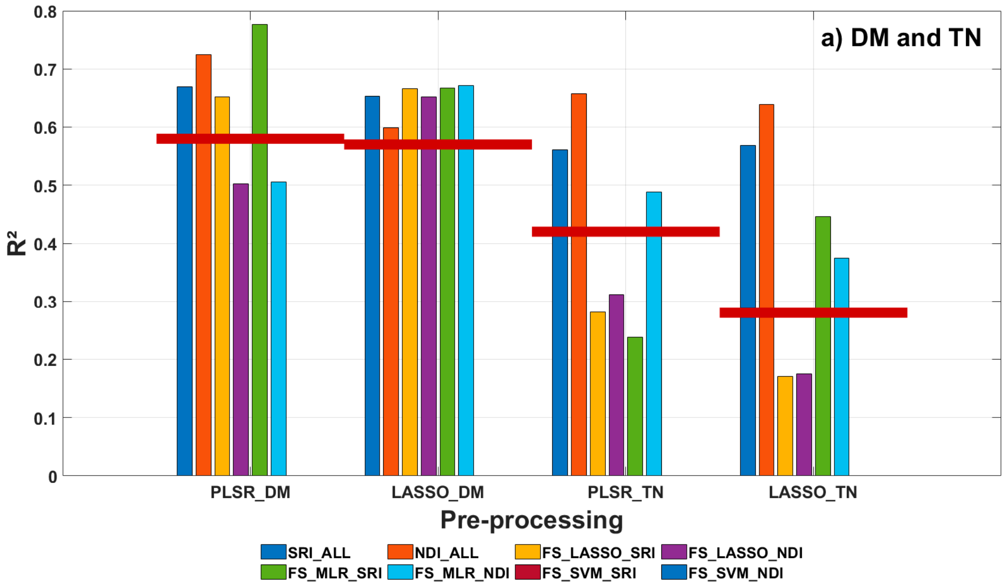

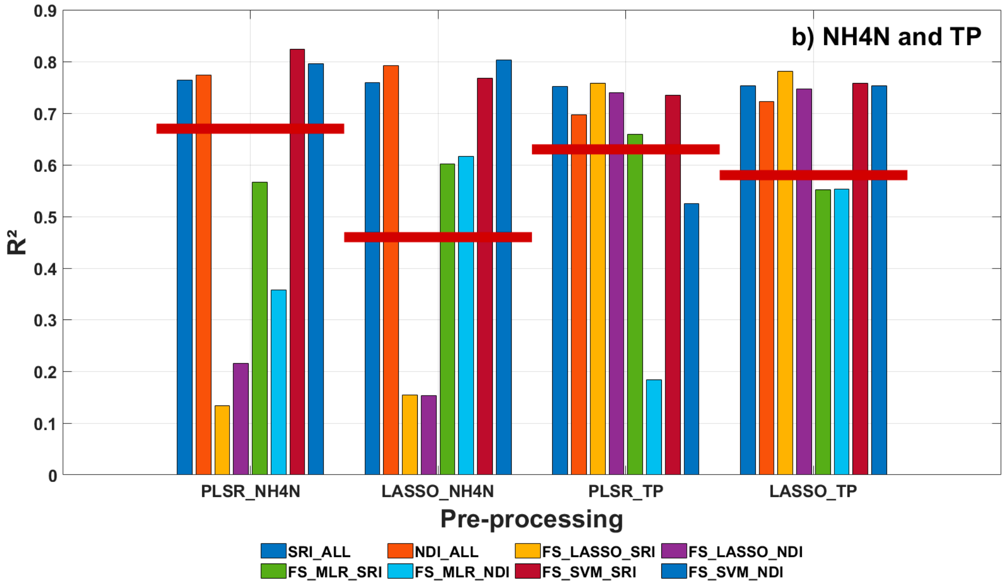

Supplementary Figure S3. The performance comparison of two-band indices transformations for predicting a) DM and TN, b) NH4N and TP is presented. The red horizontal line represents the performance of the raw data, serving as a baseline for comparison. PLSR (partial least square regression); L: LASSO (least absolute shrinkage and selection operator) regression.

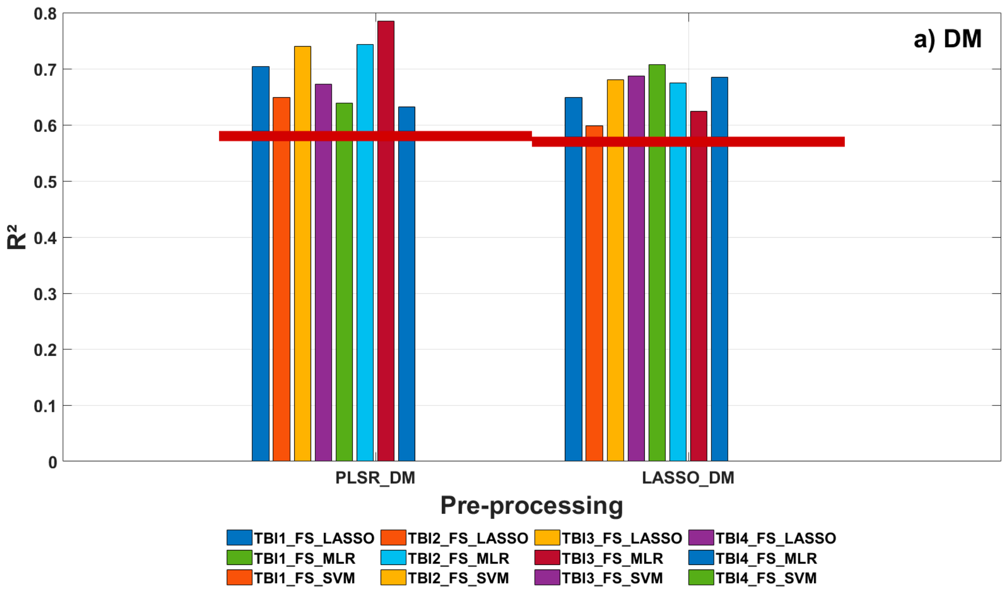
a) DM
R²
0.8
0.7
0.6
0.5
0.4
0.3
0.2
0.1
0
PLSR_DM
LASSO_DM
Pre-processing
TBI1_FS_LASSO
TBI2_FS_LASSO
TBI3_FS_LASSO
TBI4_FS_LASSO
TBI1_FS_MLR
TBI2_FS_MLR
TBI3_FS_MLR
TBI4_FS_MLR
TBI1_FS_SVM
TBI2_FS_SVM
TBI3_FS_SVM
TBI4_FS_SVM

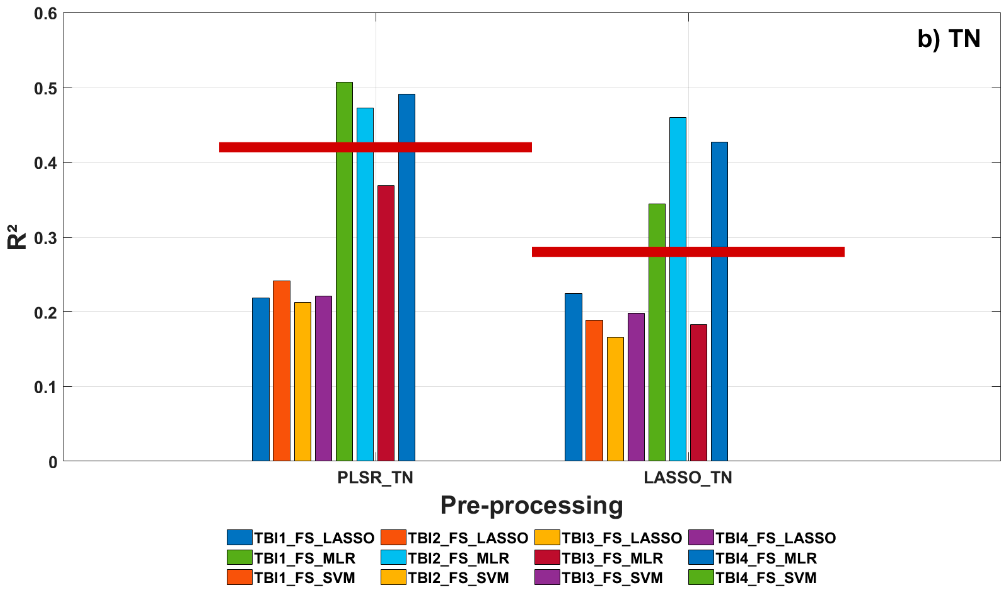
b) TN
R²
0.6
0.5
0.4
0.3
0.2
0.1
0
PLSR_TN
LASSO_TN
Pre-processing
TBI1_FS_LASSO
TBI2_FS_LASSO
TBI3_FS_LASSO
TBI4_FS_LASSO
TBI1_FS_MLR
TBI2_FS_MLR
TBI3_FS_MLR
TBI4_FS_MLR
TBI1_FS_SVM
TBI2_FS_SVM
TBI3_FS_SVM
TBI4_FS_SVM

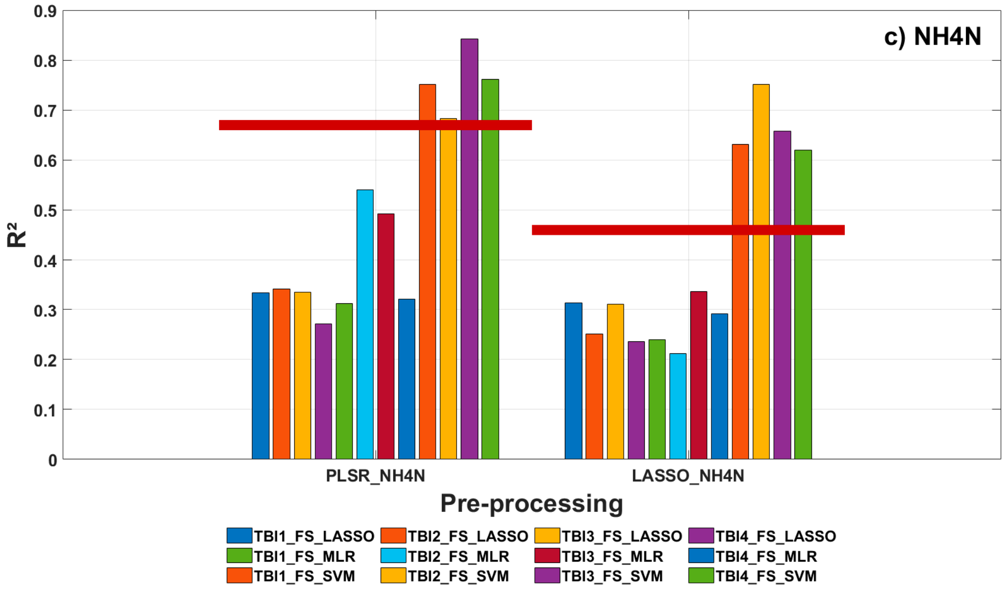

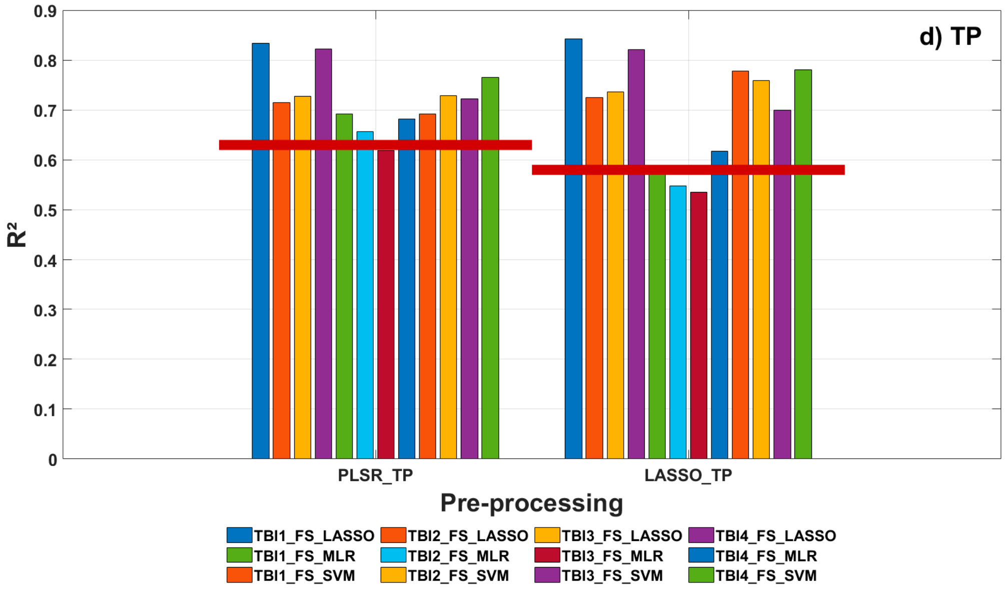

Supplementary Figure S4. The performance comparison of three-band indices transformations for predicting a) DM, b) TN, c) NH_4-N, and d) TP is presented. The red horizontal line represents the performance of the raw data, serving as a baseline for comparison.
